# Cats with Genetic Variants of AGXT2 Respond Differently to a Dietary Intervention Known to Reduce the Risk of Calcium Oxalate Stone Formation

**DOI:** 10.3390/genes13050791

**Published:** 2022-04-28

**Authors:** Jean A. Hall, Kiran S. Panickar, Jeffrey A. Brockman, Dennis E. Jewell

**Affiliations:** 1 Department of Biomedical Sciences, Carlson College of Veterinary Medicine, Oregon State University, Corvallis, OR 97331, USA; 2 Science & Technology Center, Hill′s Pet Nutrition, Inc., Topeka, KS 66617, USA; kiran_panickar@hillspet.com (K.S.P.); jeff_brockman@hillspet.com (J.A.B.); 3 Department of Grain Science and Industry, Kansas State University, Manhattan, KS 66506, USA; djewell@ksu.edu

**Keywords:** AGXT2, betaine, calcium oxalate, cats, metabolomics, personalized nutrition, urine

## Abstract

This study was completed to evaluate a genotype-specific nutritional intervention for reducing the risk of calcium oxalate stone formation. Serum metabolomic profiles and genotypes of 445 cats in the colony at Hill’s Pet Nutrition, Inc (Topeka, KS, USA)were assessed in a genome-wide association study, and revealed an association between genetic variants of alanine-glyoxylate aminotransferase 2 (AGXT2) and 2-oxoarginine. The most significant single nucleotide polymorphisms (SNP) associated with 2-oxoarginine was at position chrA1:212069607, [G/A] (*p* < 3.687 × 10^−17^). This SNP explained approximately 15% of the variance in 2-oxoarginine concentrations. The distribution of genotype frequencies was 0.07 AA, 0.39 AG, and 0.54 GG, with a mean relative 2-oxoarginine concentration for each genotype of 0.45 AA, 0.92 AG, and 1.27 GG, indicating a subtractive effect of the minor allele (A). Serum concentrations of two AGXT2 substrates, symmetric/asymmetric dimethylarginines (SDMA/ADMA) and β-aminoisobutyrate (BAIB) were also strongly associated with SNP chrA1:212069607 (*p* < 1.43 × 10^−12^ and *p* < 2.30 × 10^−14^, respectively). These two AGXT2 substrates were increased with the minor allele (A), indicating that the variant of the AGXT2 gene results in decreased aminotransferase activity. Additionally, the lifetime history of stone incidence showed that cats with the AA variant of AGXT2 SNP had a 2.515× increased incidence of stones compared with cats having the GG variant (*p* = 0.019). In a subsequent study assessing AGXT2 genotypes, cats (*n* = 10 GG, 4 AG, 9 AA) were fed control or test food (containing betaine at 0.500%, and the botanicals green tea, fenugreek and tulsi at 0.25, 0.025, and 0.0015%, respectively) in a cross-over study design. Stone risk analysis was conducted on urine samples after feeding control or test food for 28 days each. A calcium oxalate titration test (COT) was performed to assess the amount of added Ox^−2^ (per L) required to initiate calcium oxalate crystal formation. Cats with the GG variant of the AGXT2 SNP required more added oxalate to initiate urine crystal formation after consuming test food compared with control food, indicating a decreased risk of oxalate crystal formation in GG cats. In addition, urine oxalate concentrations showed an overall effect of test food independent of genotype (*p* = 0.0009), which resulted in lower oxalate concentrations after consuming test food compared with control food. These data indicate that cats with the GG-specific variant of AGXT2 should benefit from a reduced risk of calcium oxalate stone formation after consuming a betaine and botanical dietary enhancement.

## 1. Introduction

The overall goal of personalized nutrition is to optimize health and well-being using relevant information about individuals rather than the entire population [1]. Relevant information can include clinical assessments, biomarkers, and genetic information. Genetic-guided nutritional supplementation uses food as therapy to benefit certain genotypes, and could potentially counteract a negative genetic variant in a targeted and personal way [2].

Cells occasionally make mistakes when copying DNA during cell division, which in some cases leads to variations in the DNA sequence at particular locations, called single nucleotide polymorphisms, or SNPs. SNPs provide biological variation between animals by changing the recipes for proteins encoded by a gene [2]. Genetic polymorphism refers to the appearance within a population of two or more distinct genetic variants. Genetic variants can influence disease susceptibility, disease progression, and therapeutic responses [3]. Gene variants are named according to how frequently they occur: the type most widely distributed is usually the wild type (homozygous), followed by the heterozygous type, and finally, the mutation (homozygous), which is usually least abundant [4].

A genome-wide association study (GWAS) is a big-data approach used to associate specific genetic variants with particular diseases or other response variables. It involves scanning the genome of many individuals to find genetic variations associated with a particular disease or biomarker. A large number of subjects are needed in order to obtain a reliable signal, given the very large number of tests that are required. Thus, associations must show a high level of significance to survive the multiple testing correction. Such studies are very useful for finding genetic variations that contribute to common, complex diseases, and provide the scientific basis for a personalized nutrition plan to lessen the effects of the identified genetic predisposition [2].

We have previously reported that the lifespan of cats with non-obstructive kidney stones is shortened compared with healthy cats indicating a need to prevent stone formation and minimize chronic kidney disease [5]. The majority of stones in cats are calcium oxalate or magnesium ammonium phosphate (struvite) uroliths. Of the two, it is generally accepted that medical dissolution is not possible for calcium oxalate uroliths, whereas struvite uroliths in cats can be dissolved in 1 to 4 weeks by feeding specially designed food [6]. Using a urine calcium oxalate titrimetric test (COT), we have shown a benefit for reducing oxalate crystal formation in cats by increasing dietary polyunsaturated fatty acids (PUFA) [5]. Because the COT test is based on adding calculated amounts of oxalate to urine in vitro and quantitating when calcium oxalate crystal formation begins, we are also interested in determining how excess oxalate is secreted into urine in vivo to cause calcium oxalate stone formation.

Primary hyperoxalurias are rare inborn errors of glyoxylate metabolism resulting from excessive oxalate production, mainly in the liver. Oxalate must be eliminated by filtration at the glomerulus or secretion by the renal tubules [7]. There are two alanine-glyoxylate aminotransferase (AGXT) isoenzymes: AGXT1 and AGXT2. Both transfer an amino group from alanine to glyoxylate. Glyoxylate is a two-carbon keto-acid formed as an intermediary from glycine, hydroxyproline, and glycolate metabolism [7]. It is readily catalyzed by several dehydrogenases and oxidases, including lactate dehydrogenase (LDH), which can convert it into oxalate, an end product in mammalian metabolism that has to be eliminated in the urine [7]. Most of the glyoxylate generated, however, is further metabolized within organelles such as the peroxisome and mitochondria to limit oxalate production. Glyoxylate in the cytosol and mitochondria can also be made into highly soluble glycolate by cytosolic glyoxylate reductase (GRHPR) [7]. Glycolate is then further metabolized in peroxisomes to make glycine, after first being re-oxidized to glyoxylate by glycolate oxidase (GO). The peroxisome membrane is readily permeable to glycolate and glyoxylate, which shields the surrounding cytoplasm from glyoxylate accumulation and secondary oxalate production [7].

In humans, at least 178 mutations in the gene encoding AGXT have been described [8]. Most of these mutations decrease or eliminate the activity of AGXT, which decreases the conversion of glyoxylate to glycine, and results in hyperoxaluria. Oxalate is excreted in the urine as a waste product, or combines with calcium in the urine to form calcium oxalate crystals. Genotyping in humans has revealed at least six AGXT2 coding SNPs [9], including rs37369 (c.418 G>A, p.Val140Ile) [10].

The presence of AGXT2 SNPs in cats and their association with oxalate crystal formation in urine has not been well studied. Furthermore, personalized nutrition to benefit specific AGXT2 genotypes has not been previously reported. The purpose of this study was to evaluate the systemic effects of two diets (control and test diet enriched with betaine and botanicals) in cats with different AGXT2 genetic variants. We chose a combination of ingredients that we thought would be synergistic in improving urinary tract health, changing fractional excretion of calcium, changing inflammation and hopefully reducing the risk of calcium oxalate stone formation. We had previously published a study using this same botanical package in cats and showed that pro-inflammatory cytokines MCP-1, TNFα, SDF-1, Flt3L, IL-8, IL-12p40, IL-13, and IL-18 were all reduced (*p* < 0.05) [11].

## 2. Materials and Methods

All study protocols were reviewed and approved by the Institutional Animal Care and Use Committee, Hill’s Pet Nutrition, Inc., Topeka, KS, USA (Permit Number: CP815.1.2.0-A-F-D-ADH-MULTI-84-KID), and complied with the National Institutes of Health Guide for the Care and Use of Laboratory Animals [12]. Cats were housed in groups and allowed access to indoor runs. Cats also had exposure to natural light that varied with seasonal changes. All cats were provided with regular opportunities to exercise, with access to toys. Cats were owned by the commercial funders of this research or their affiliates, who gave permission for them to be included in this study. At the conclusion of the study, all cats were returned to the Hill’s Pet Nutrition, Inc. colony.

### 2.1. Cat Colony and Feeding Study Participants

Metabolomic profiles (Metabolon, Morrisville, NC, USA) and genotyping of 445 cats (random bred) in the colony at Hill’s Pet Nutrition, Inc. were performed for a GWAS study. The cats were genotyped on a custom cat iSelect Illumina high-density genotyping array with 340,000 attempted bead types. Approximately 272,000 SNPs gave reliable genotype calls. Genotypes were converted to population-based linkage analysis (PLINK) format and quality control was performed in PLINK v1.9 (https://www.cog-genomics.org/plink2, accessed on 1 March 2022) [13,14]. After filtering (genotype calls > 0.90, maf > 0.05), 437 individuals and 194,151 SNPs remained in the data set. The relative normalized concentrations of serum metabolites were used as a continuous variable for quantitative trait loci (QTL) analysis using an additive linear model with the first two eigenvectors of a principal component analysis (PCA) of the cohort to identify loci associated with serum metabolite concentrations. *p* values, r^2^ values, and mean serum concentrations for each genotype were calculated for the SNP A3:212069607 association with 2-oxoarginine, dimethylarginine, and β aminoisobutyrate (BAIB) using the –assoc qt-means tag in PLINK.

A subsequent study used 23 cats of three genotypes defined by the single nucleotide polymorphism at the AGXT2 gene. There were 10 GG cats, 9 AA cats, and 4 GA cats. All cats were of domestic shorthair breed, and either spayed females (*n* = 13) or neutered males (*n* = 10). At baseline, cats were adults with an average age of 6.1 years (range 2 to 10 years). Body weights ranged from 3.6 to 6.1 kg. These cats were determined to be healthy based on the results of an annual physical examination, CBC, serum biochemistries, and urinalysis. Inclusion criteria were cats with known GG, GA, or AA genotypes. Cats were excluded from the study if they were known to have problems eating new foods or were irritable about repeat blood sampling, and/or had any other diagnosed disease condition such as diabetes, cancer, inflammatory bowel disease, dermatitis, or food allergy. The criterion for removal from the study was the development of any condition whereby removal would benefit the animal, including any cat refusing to eat, or inadequate food intake resulting in weight loss greater than 15% of body weight. No cats were removed from the study.

### 2.2. Personalized Nutrition Study Design

**Figure 1:**
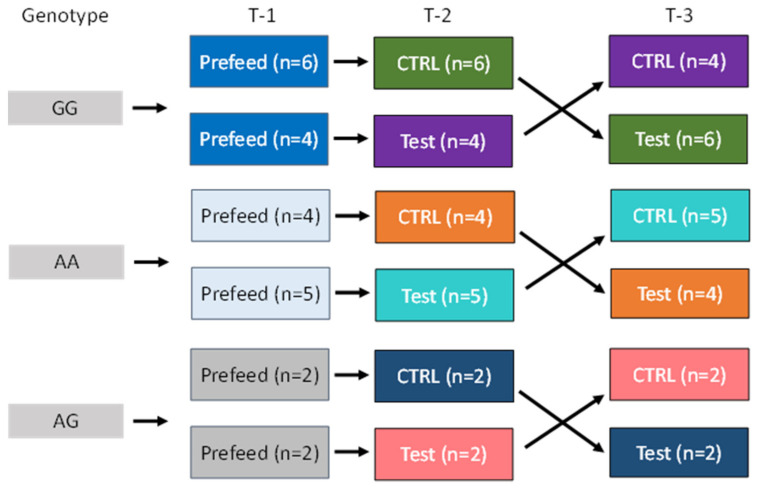
Cats with three alanine-glyoxylate aminotransferase 2 (AGXT2) single nucleotide polymorphisms (SNPs: GG, AG, and AA) were fed control food for 28 days (Prefeed; T-1). Cats within each genotype were then split into two groups and fed control (CTRL) or test food in a cross over study design for 28 days each (T-2 and T-3).

Cats with three AGXT2 genotypes were fed control or test food in a crossover feeding study design (Figure 1). Pre-trial, all cats were fed control food for 28 days. Cats within each genotype were then split into two groups. Each group of cats was then fed one of two foods (control or test food) for 28 days, after which time the cats were crossed over to the opposite food and fed for 28 days. The 28-day feeding period was chosen based on a previous study whereby we learned that we could find a difference, if one existed, after a 28-day feeding period [5]. In that study, after consuming test food, cats had increased (all *p* < 0.001) serum concentrations of EPA (173%), DHA (61%), and AA (35%); decreased urine specific gravity (*p* = 0.02); decreased urine calcium concentration (*p* = 0.06); decreased relative-super-saturation for struvite crystals (*p* = 0.03); and increased resistance to oxalate crystal formation (*p* = 0.06) compared with cats consuming control food at 28 and 56 days.

The pre-trial food (control food) was a complete and balanced dry food designed to aid in the management of renal stone disease. Test food was the pre-trial food supplemented with betaine (0.500%) and botanicals [green tea (0.25%), fenugreek (0.025%) and tulsi (0.0015%)]. All cats had access to electronic feeders whereby fresh food was offered daily with amounts available for consumption calculated to maintain body weight; water was available *ad libitum*. Food intake was monitored throughout the study.

Blood and urine samples were collected at baseline (end of pre-trial period) and at the end of each 28-day feeding period. Blood assays included CBC, selected serum biochemistries and cytokines, and analysis of serum metabolomic profiles. Urine assays included urinalysis, urine protein/creatinine (UPC) ratio, and assays for stone risk analysis. Bodyweight and composition were measured using a dual-energy X-ray absorptiometry (DEXA) scan at baseline and after each feeding period.

### 2.3. Foods

A complete and balanced dry food designed to aid in the management of renal calculi, was fed during the pre-trial period and was the control food. Test food was the pre-trial food supplemented with betaine (included at 0.500%), and botanicals [green tea (0.25%), fenugreek (0.025%) and tulsi (0.0015%)]. Both foods (Table 1) were prepared by Hill’s Pet Nutrition, Inc., and met the nutritional requirements for adult cats (≥1 year) as established by the Association of American Feed Control Officials (AAFCO). Food was available in dry form only. Macronutrient composition and fiber concentrations of foods were determined by a commercial laboratory (Eurofins Scientific, Inc., Des Moines, IA, USA). Proximate analyses were completed using the following techniques: moisture—AOAC 930.15; protein—AOAC 2001.11; fat—AOAC 954.02; fiber—AOAC 962.09; and ash—AOAC 942.0. Mineral and fatty acid analyses were performed by the same commercial laboratory. Fatty acid (FA) concentrations were determined by gas chromatography of FA methyl esters, and were expressed as g/100 g of FAs as fed. The sum of dietary saturated FA (SFA) was determined as follows: 8:0 + 10:0 + 11:0 + 12:0 + 14:0 + 15:0 + 16:0 + 17:0 + 18:0 + 20:0 + 22:0 + 24:0. The sum of dietary monounsaturated FA (MUFA) was determined as follows: 14:1 + 15:1 + 16:1 + 17:1 + 18:1 + 20:1 + 22:1 + 24:1. The sum of dietary polyunsaturated FA (PUFA) was determined as follows: 18:2 (n-6) + 18:3 (n-6) + 18:3 (n-3) + 18: 4 (n-3) + 20:2 (n-6) + 20:3 (n-6) + 20:3 (n-3) + 20:4 (n-6) + 20:4 (n-3) + 20:5 (n-3) + 21:5 (n-3) + 22:2 (n-6) + 22:4 (n-6) + 22:5 (n-6) + 22:5 (n-3) + 22:6 (n-3).

Both foods contained similar concentrations (within analytical variance of targets) of protein, and had similar predicted caloric content. Likewise, crude fiber and fatty acid composition were similar for control and test foods. Ingredients were the same in both foods (by order of preponderance: chicken, corn, corn gluten meal, wheat, rice, chicken meal, pork fat, soybean oil, fish oil). Rice was reduced from the control food to allow for the addition of 0.500% betaine, and botanicals [green tea (0.25%), fenugreek (0.025%) and tulsi (0.0015%)]. Both foods received similar amounts of vitamins, amino acids and minerals.

### 2.4. Serum and Urine Analyses

Blood was collected from each cat (after withholding food for 17 h; food was withheld at the end of the day and blood was drawn the next morning) at each time point (baseline and at the end of each 4-week feeding period) to assess complete blood count and serum chemistries. Serum cytokines were assessed by ELISA at each time point. Total T4 and blood taurine concentrations were assessed only at baseline to rule out underlying thyroid disease or deficiency.

Urine was collected by cystocentesis and submitted for immediate analysis of urine pH, urine specific gravity (USG), routine dipstick analysis, semi-quantitative urine sediment analysis, and calculation of urine protein: creatinine (UPC) ratio. Urine-specific gravity was determined using a refractometer. Urine creatinine concentration is used to eliminate the effect of the concentration status of urine, and measured with the same assay as serum creatinine. Urine protein concentrations were determined using urine supernatant (benzethonium chloride turbidometric method). The UPC ratio calculations were determined as previously reported [16] and expressed as mg/dL protein:mg/dL creatinine. Urine calcium concentration was measured with the same assay as serum calcium. Urine oxalate concentration was measured by ion exclusion chromatography after solid-phase extraction as previously published [17].

Stone risk analysis was completed on urine samples for struvite relative super saturation (RSS) and for a calcium oxalate titration test (COT). The analysis for struvite crystals was performed using the EQUIL 2 program [18,19,20]. In brief, this computer program calculates a urine supersaturation ratio (unitless) with respect to the common kidney stone components. The EQUIL 2 program provides an evaluation of the state of urinary saturation based on pH and total concentrations (M/L) of specific analytes. This program uses sodium, potassium, calcium, magnesium, chloride, ammonium, citrate, phosphate, sulfate, and oxalate concentrations. The method also uses thermodynamic stability constants to calculate free ion activities for urinary ions. These free ion activities are then used to calculate the supersaturation ratio of urine compared with what would form crystals in pure water.

The COT test was performed using a method adapted from Laube et al. [21,22,23]. It is also called the Bonn-Risk Index in humans. In brief, the [Ca^2+^]/[added Ox^2−^] ratio is calculated (per liter). The ratio represents the concentration of ionized calcium in the urine and the amount of oxalate that is added to initiate crystallization. An increasing index value denotes samples at greater risk of calcium oxalate crystallization, whereas decreasing index values denotes those with less risk.

### 2.5. Serum Metabolomics for Feeding Study Cats

Analysis of serum metabolomic profiles was performed by a commercial laboratory (Metabolon, Morrisville, NC, USA) as previously described [24]. Briefly, extracted supernatant was split and run on gas chromatography and liquid chromatography mass spectrometer platforms in randomized order. Gas chromatography (for hydrophobic molecules) and liquid chromatography (for hydrophilic molecules) were used to identify and provide relative quantification of small metabolites present in plasma and urine samples. Endogenous biochemicals included amino acids, peptides, carbohydrates, lipids, nucleotides, cofactors and vitamins. The complete dataset is shown as a heat map of statistically significant biochemicals profiled in this study (Table S1).

### 2.6. Statistical Methods

Statistical analyses were performed in SAS version 9.4 (SAS Institute, Cary, NC, USA) for food intake and body weights. These data were normally distributed. The CBC, serum chemistries, and urinalyses data were analyzed using an ANOVA mixed model. Some data were natural log transformed before analysis (struvite RSS, COT test). Metabolomics data were analyzed using Array Studio (OmicSoft Corporation, Cary, NC, USA). Matched-paired *t* tests were used to compare means taken on each cat after the consumption of test and control foods. Significance was established when *p* ≤ 0.05 (for type 1 error) and *q* ≤ 0.1 (*q*-values were used to estimate false discovery rates in multiple comparisons.) A chi-squared test was used to compare observed results with expected results for incidence of stones in cats with the AA variant of AGXT2 SNP compared with GG cats.

## 3. Results

### 3.1. Genome Wide Association Study of Colony Cats

A GWAS of 445 cats in the colony at Hill’s Pet Nutrition, Inc. revealed a 1.6 megabase region on cat chromosome A1 (chrA1) with a strong association to serum concentrations of the metabolite 2-oxoarginine, which is produced by the deamination of arginine. The coding sequence for the AGXT2 gene is centered in this region (located between base pair positions 210,631,461 to 212,243,690 [NCBI Felis Catus genome assembly 6.2/felcat5]). The most significant SNP associated with 2-oxoarginine in this region was at position chrA1:212069607, [G/A] (*p* < 3.687 × 10^−17^). This SNP explains approximately 15% of the variance in 2-oxoarginine serum concentrations in this cohort of cats. The distribution of genotype frequencies across the cohort of cats was 0.07 AA, 0.39 AG, and 0.54 GG, with a mean relative serum 2-oxoarginine concentration for each genotype of 0.45 AA, 0.92 AG, and 1.27 GG, indicating an additive effect of the minor allele (A) (Table 2).

Further analysis revealed that serum concentrations of two AGXT2 substrates, SDMA/ADMA dimethylarginines and β-aminoisobutyrate (BAIB) were also strongly associated with SNP chrA1:212069607 (*p*< 1.43 × 10^−12^, and *p*< 2.30 × 10^−14^, respectively; Table 2). These two AGXT2 substrates are increased with the minor allele (A), indicating that the variant of the AGXT2 gene results in decreased aminotransferase activity.

Other notable findings in the 445 colony cats were the lifetime history of stone incidence (based on ultrasound findings in live cats evaluated routinely, and necropsy findings in cats that died) on a per cat basis of stones (combined kidney and urinary tract). Cats with the AA variant of AGXT2 SNP had a 2.515 × increased incidence of stones compared with cats having the GG variant (*p* = 0.019). Cats with the AG genotype had an incidence somewhat in between at 1.49 × greater than cats having the GG variant.

### 3.2. The Effect of Dietary Treatment on Food Intake and Body Weights

Food intake and body weight were unchanged by dietary treatment (Table 3).

### 3.3. The Effect of Dietary Treatment on CBC and Selected Serum Chemistries

Normal CBC and serum chemistries were maintained in cats of all genotypes after consuming both foods. Triglyceride concentrations in cats with the AGXT2 SNP AA decreased after consuming test food compared with control food (Table 4). Triglyceride concentrations also were significantly higher in cats with the AG SNP after consuming test food compared with cats that had AA or GG SNPs after consuming test food.

There was a significant (*p* < 0.05) multivariate effect of genotype for multiple cytokines, although there was no effect of food and no interaction between food and time (Table 5). Overall, cats with the GG genotype had a more pro-inflammatory cytokine profile than cats with the AA genotype. The cytokines IL-1β, IL-6, IL-13, TNF-α, keratinocyte chemoattractant (KC), RANTES, interferon-γ, stem cell factor, and FS-7-associated surface antigen all had lower cytokine concentrations in cats with the AA genotype compared with cats having the GG genotype.

### 3.4. The Effect of Dietary Treatment on Urinalysis Parameters

Urine creatinine and urine calcium concentrations were not significantly affected by food or time, although calcium concentrations were numerically lower in GG cats after consuming test food and numerically higher in AA cats (Table 6). The range of urine calcium in GG cats over time was 14.7 to 59.4 mg/dL, whereas the range in AA and AG cats over time was 13.2 to 72.2 mg/dL. Urine oxalate concentrations were not significantly affected by genotype. Urine oxalate concentrations ranged from 471 to 1435 µM in GG cats over time and from 366 to 1691 µM in AA and AG cats. However, there was an overall effect of test food independent of genotype (*p* = 0.0009), which resulted in lower oxalate concentrations after consuming test food compared with control food.

There was not a significant effect of food or time on urine pH (Table 6). Urine pH was numerically higher in GG cats fed both foods compared with baseline, and numerically higher in GG cats at all times compared with AA or AG cats. The range of urine pH in GG cats over time was 5.85 to 7.27, whereas the range in AA and AG cats over time was 5.53 to 6.62. The USG was also not affected by food, time, or AGXT2 SNP variant.

Fractional excretion of calcium (%) was calculated as urine calcium concentration/serum calcium concentration divided by urine creatinine concentration/serum creatinine concentration × 100. There was not a significant effect of food or time on fractional excretion of calcium (Table 6). Fractional excretion of calcium was numerically increased from baseline in AA cats fed test food, but numerically decreased in GG cats fed test food. The range of fractional excretion values in AA cats fed test food was 0.07 to 0.21%, whereas the range in GG cats fed test food was 0.06 to 0.18%. Using fractional excretion of calcium (*p* < 0.001) and USG (*p* = 0.018) measurements (interaction was not significant), we could predict risk of oxalate crystal formation (r^2^ = 0.76). The coefficient of determination was increased further if pH and all interactions were considered (r^2^ = 0.90).

Stone risk analysis for struvite RSS was not significantly affected by food, time, or genetic variants for AGXT2 (Table 6).

**Figure 2:**
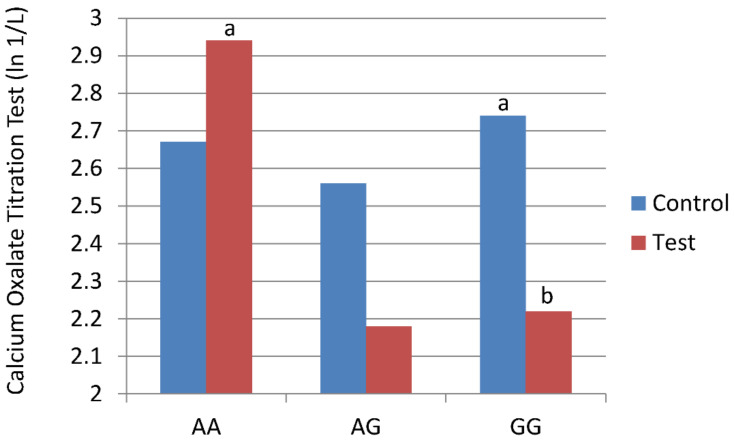
Cats with three alanine-glyoxylate aminotransferase 2 (AGXT2) single nucleotide polymorphisms (SNPs: GG, AG, and AA) were fed control food or test food for 28 days in a cross-over study design. Stone risk analysis for calcium oxalate crystal formation, assessed using the calcium oxalate titrimetric (COT) test, showed that cats with the GG variant of the AGXT2 SNP had lower values (decreased susceptibility to added oxalate before forming calcium oxalate crystals) after consuming test food compared with control food. ^a,b^ Means with different superscripts are different (*p* < 0.05; largest Std Error = e ^0.40^).

Stone risk analysis for calcium oxalate crystal formation, assessed using the COT test, showed that cats with the GG variant of the AGXT2 SNP had lower values (decreased susceptibility to added oxalate before forming calcium oxalate crystals) after consuming test food compared with control food (Table 6). Thus, feeding test food decreased the risk of oxalate crystal formation. The COT test values were significantly higher in cats with the AA SNP after consuming test food compared with cats that had the GG SNP after consuming test food (Figure 2).

### 3.5. The Effect of Dietary Treatment on Serum Metabolites

Relative serum metabolite concentrations were compared for each AGXT2 SNP variant after feeding control food or test food for 4 weeks in a crossover feeding trial (Table 7). For each metabolite, the mean value is the group mean when data is re-scaled to have a medial equal to 1. Values are the ratio of concentrations after eating test food divided by concentration after eating control food. Green denotes a decline and red an increase in the ratio (test food concentration/control food concentration) of each metabolite.

In this study, 818 named biochemicals were detected in feline serum. Analysis of these biochemicals revealed a differential effect of AGXT2 SNP variants on the metabolome with GG cats having more analytes that changed in a significant fashion (*n* = 83; 15 increased and 68 decreased) as compared to AA cats (*n* = 43; 9 increased and 34 decreased) or AG cats (*n* = 28; 27 increased and 1 decreased). 

Consumption of test food containing betaine resulted in higher serum concentrations of betaine in cats of all genetic variants. Betaine functions as a methyl donor for the synthesis of methionine from homocysteine, and in the process, betaine is converted to dimethylglycine, which is further metabolized to sarcosine. Sarcosine was also increased in cats of all genetic variants after consuming test food. Other test food components showed a similar increase in serum, e.g., theophylline and eugenol sulfate.

There was a genotype-specific difference in fatty acid metabolism. The lysophospholipids 1-palmitoyl-GPE (16:0), 1-stearoyl-GPE (18:0), 1-arachidonoyl-GPE (20:4n6), and 1-(1-enyl-stearoyl)-2-oleoyl-GPE (P-18:0/18:1), and the diacylglycerol oleoyl-linoleoyl-glycerol (18:1/18:2) were decreased in cats with the AA genotype compared with cats having the GG genotype after consuming control or test food for 28 days.

Several serum metabolites of *a priori* interest are shown in Table 8. These are scaled imputed values (not log transformed) of the concentrations of selected genotype-specific serum metabolites. Values were significantly higher for 2-oxoarginine, S-adenosylhomocysteine, and cortisol in cats with the GG genotype compared with cats having the AA genotype. Conversely, values were significantly higher for BAIB and 9-HODE + 13-HODE in cats with the AA genotype compared with cats having the GG genotype. There was a significant genotype × food interaction for the oxidized glutathione metabolite. The values for S-adenosylhomocysteine were higher in cats with the GG and AG genotypes after consuming test food, whereas values for oxidized glutathione were lower in cats with the GG genotype after consuming test food compared with control food. Values of serum oxalate concentrations were not significantly different based on genotype or food consumption (numerically decreased).

## 4. Discussion

A strong association between serum 2-oxoarginine concentrations and a region of chromosome A1 was found in this study. A SNP at position chrA1:212069607, [G/A], exhibited genotype frequencies across the cohort of cats of 0.07 AA, 0.39 AG, and 0.54 GG. The mean relative 2-oxoarginine concentration for each genotype was 0.45 AA, 0.92 AG, and 1.27 GG, indicating a negative association of the minor allele (A) with 2-oxoarginine concentrations. The 2-oxoarginine metabolite is produced by the deamination of arginine. Because this SNP is in the region of the AGXT2 gene, genetic variants are likely to negatively affect AGXT2 activity, similar to human AGXT2 SNPs [10,25]. In humans, AGXT2 activity has been found only in mitochondria in all mammalian species tested. Human AGXT2 is encoded by a gene located on chromosome 5. After synthesis in the cytoplasm, AGXT2 is transported to mitochondria where modification of the N-terminus leads to the formation of the mature enzyme. AGXT2 is expressed primarily in the kidney and liver [26].

AGXT2 metabolizes both ADMA and SDMA, as well as BAIB, the latter being an end product of pyrimidine metabolism (reviewed in [10]). In our GWAS study, serum concentrations of the two AGXT2 substrates, the SDMA/ADMA dimethylarginines, and BAIB were strongly associated with SNP chrA1:212069607. These two AGXT2 substrates were increased with the minor allele (A), indicating that the variant of the AGXT2 gene results in decreased aminotransferase activity.

Another major finding of this study was that feeding test food containing betaine and botanicals resulted in decreased urine oxalate concentrations in all cats regardless of genetic variants of the AGXT2 gene. Nonetheless, only cats with the GG variant of the AGXT2 gene actually had a statistically significant decrease in COT ratios. This ratio is based on the concentration of ionized calcium in the urine and the amount of oxalate that must be added to initiate crystallization. A decreased ratio [Ca + 2]/[added Ox-2] means more oxalate has to be added to initiate crystallization. In the GG cats, a decreased ratio means they have less risk of oxalate stone formation compared with AA cats that have a higher COT ratio. Although food reduced oxaluria regardless of the genotype, we note this finding secondarily, because it appears that urine calcium concentration is more important than urine oxalate concentration in determining crystal formation. Nonetheless, a diet that reduces oxalate concentrations in all cats is important.

These findings with the COT test ratio were consistent with phenotype findings in colony cats, in that cats with the AA variant of AGXT2 SNP had a 2.515 x increased lifetime incidence of stones compared with cats having the GG variant. The risk of calcium oxalate stone formation in cats has not been previously linked with genotype, specifically genetic variants of AGXT2.

In humans, the majority of glyoxylate metabolism results from AGXT1 rather than AGXT2 activity [27]. Impairment of glyoxylate metabolism leads to increased oxidation of glyoxylate to oxalate and deposition of insoluble calcium oxalate crystals in the urinary tract. This occurs despite intact AGXT2 expression and activity, which suggests that the metabolism of glyoxylate by AGXT2 cannot compensate for the absence of AGXT1 (reviewed in [27]). It is possible that the substrate specificity of AGXT2 is strongly pH-dependent, although it isn’t known whether physiological changes in pH in the mitochondrial matrix are sufficient to influence substrate specificity of AGXT 2 (reviewed in [27]). Thus, it is unclear why colony cats with altered AGXT2 activity historically have more calcium oxalate stones.

Cats with the AA genetic variant of AGXT2 had significantly higher BAIB concentrations in the feeding study compared with cats having the GG genetic variant, similar to what was noted in the GWAS. With a larger number of cats in the GWAS, the dimethyl arginines (SDMA + ADMA) were also increased in cats with the AA variant. In the feeding study, the dimethyl arginine concentrations were numerically higher, but not significantly different in cats with the AA variant compared with the GG variant. Hyper-β-aminoisobutyric aciduria in humans has also been associated with the AGXT2 rs37369 polymorphism located on chromosome 5p13 [28]. Other AGXT2 polymorphisms have also been reported to exhibit elevated BAIB concentrations in plasma and urine, suggesting that AGXT2 plays a physiological role in regulating BAIB concentrations in humans (reviewed in [27]). Interestingly, elevated plasma BAIB has been associated with decreased concentrations of serum triglycerides [29], similar to findings in our study whereby triglyceride concentrations were highest in cats with the AG genetic variant. In our study, feeding betaine enriched test food to cats with the AA genetic variant resulted in lower triglyceride concentrations. It is uncertain the effect of BAIB on lipid homeostasis in general (reviewed in [27]), but we also saw genotype-specific differences in fatty acid metabolism. In particular, the lysophospholipids were decreased in cats with the AA genotype compared with cats having the GG genotype after consuming control or test food for 28 days.

The AGXT2 rs37369 polymorphism has been associated with increased SDMA levels in the plasma and urine of healthy human volunteers [10]. At least in mice, AGXT2 can metabolize ADMA at physiological concentrations [30] to form α-keto-δ-(NG,NG-dimethylguanidino)valeric acid (DMGV), which suggests that AGXT2 contributes to the regulation of plasma levels of methylarginines (reviewed in [27]). We have also shown here that cats with the AA genetic variant had increased SDMA/ADMA dimethylarginine concentrations. It is possible that increased plasma dimethylarginine concentrations occur secondary to increased BAIB concentrations because of competitive inhibition of AGXT2 activity toward methylarginines, as has been shown in mice after systemic infusion of BAIB [31].

Betaine is an important nutrient that can support methyl donation reactions to provide protective biochemicals for stressful conditions. Because test food was supplemented with 0.500% betaine, it was not surprising that betaine was increased in plasma after consumption of test food. This provided enhanced capacity for one-carbon metabolism, evidenced by increased sarcosine. Betaine functions as a methyl donor for the synthesis of methionine from homocysteine, and in the process, betaine is converted to dimethylglycine, which is further metabolized to sarcosine. Sarcosine was also increased in cats of all genetic variants after consuming test food. Increased methionine concentrations theoretically lead to increased S-adenosylmethionine (SAM) and S-adenosylhomocysteine (SAH) concentrations [32]. In our feeding study, significant differences in SAH concentrations were noted as expected with betaine-containing test food consumption, but also between genetic variants of AGXT2 (shown as a lack of response to betaine supplementation).

Decreased markers of collagen degradation, e.g., trans-4-hydroxyproline, were also observed in all cats consuming test food. Collagen is the most abundant protein in the extracellular matrix, and free trans-4-hydroxyproline is almost exclusively found in collagen. The degradation of collagen by the enzyme matrix metalloproteinases gives rise to this metabolite. Decreased levels of trans-4-hydroxyproline, thus, may reflect decreased extracellular matrix breakdown [33]. The reduction of plasma trans-4-hydroxyproline in cats implies improved collagen integrity.

Other notable findings were in the concentrations of cytokines, cortisol, and FFA-derived mediators of inflammation. Although there was no effect of food in the feeding study, cats with the GG genotype in the GWAS had a more pro-inflammatory cytokine profile compared with AA cats. The cytokines IL-1β, IL-6, IL-13, TNF-α, keratinocyte chemoattractant (KC), RANTES, interferon-γ, stem cell factor, and FS-7-associated surface antigen cytokine concentrations were higher in cats having the GG genotype. Linoleic acid, an n-6 FA, gives rise to hydroxyoctadecadienoic acids (9 and 13 HODEs), which are stable oxidation products. The generation of 9 and 13 HODEs is increased when oxidative stress is increased [34]. Oxidized glutathione was lower in cats with the AA genotype, but also lower in cats with the GG genotype after consuming test food compared with control food. Cortisol concentrations were higher in cats with the GG genotype compared with cats having the AA genotype. It is possible that the more pro-inflammatory cytokine profile in cats with the GG genotype is responsible for higher cortisol concentrations compared with cats having the AA genotype. This could result in a significant reduction in immune competence as increased cortisol is associated with reduced immune activation [35,36]. Cortisol concentrations in cats with the GG genotype, at least in this small group of cats, were not significantly lowered after consumption of test food.

The limitations of this research are the relatively small group of cats used in the feeding study. Future clinical studies are needed to determine whether increased dietary long-chain polyunsaturated fatty acids [5] in addition to a betaine and botanical dietary enhancement further lowers the risk of urine stone formation in GG cats. Finally, prospective clinical studies are needed to determine whether the betaine and botanical dietary supplement are effective for preventing actual calcium oxalate stone formation in cats.

## 5. Conclusions

Our findings show that cats have an AGXT2 coding SNP at position chrA1:212069607, [G/A], analogous to human AGXT2 SNPs. Serum concentrations of two AGXT2 substrates, SDMA/ADMA dimethylarginines and BAIB, are strongly associated with SNP chrA1:212069607 (*p* < 1.43 × 10^−12^, and *p* < 2.30 × 10^−14^, respectively). Because these two AGXT2 substrates were increased with the minor allele (A), this suggests that the AA SNP results in decreased aminotransferase activity. Cats with the AA variant of AGXT2 SNP have a 2.515 × increased incidence of stones compared with cats having the GG variant. Thus, personalized nutrition to decrease stone formation is needed. Our results suggest that a betaine and botanical dietary enhancement lowers urine oxalate concentrations independent of genotype. Furthermore, cats with the GG variant of the AGXT2 SNP required more added oxalate to initiate urine crystal formation after consuming test food compared with control food. At least in GG cats, this indicates a decreased risk for oxalate crystal formation with a betaine and botanical dietary enrichment.

## 6. Patents

United States Patent No. US 11,155,856 B2; Date of Patent 26 October 2021. Methods for identifying a companion animal susceptible to treatment that reduces the risk of stone formation and compositions for reducing such risk. Applicant: Hill’s Pet Nutrition, Inc. Inventors: Dennis Jewell, Jeffrey Brockman, Kiran Panickar, and Laura Morgan.

## Figures and Tables

**Table 1:** Composition of pre-trial (control food) ^1^ and test food ^2^.

Nutrient ^3^	Pre-Trial & Control Food	Test Food
Moisture	5.47	5.81
Protein	32.9	33.0
Fat	14.6	14.5
Atwater Energy, ^4^ kcal/kg	3815	3816
Ash	4.89	4.69
Crude Fiber	1.5	1.2
Betaine (mg/kg)	344	5210
Calcium	0.69	0.71
Phosphorus	0.80	0.83
Sodium	0.38	0.41
Magnesium	0.08	0.08
ARA [20:4 (n-6)]	0.09	0.10
EPA [20:5 (n-3)]	0.15	0.16
DHA [22:6 (n-3)]	0.11	0.12
SFA ^5^	3.40	3.28
MUFA ^6^	4.56	4.74
PUFA ^7^	3.78	3.94
Total FA	11.62	12.07
(n-6) FA ^8^	3.19	3.29
(n-3) FA ^9^	0.51	0.56
(n-6):(n-3) ratio	6.2	5.9

^1^ Pre-trial (control) food was a complete and balanced dry food designed to aid in the management of renal calculi. ^2^ Test food was prepared by Hill’s Pet Nutrition, Inc. and was similar to the pre-trial food, with the exception that it was supplemented with betaine (0.500%) and botanicals [green tea (0.25%), fenugreek (0.025%) and tulsi (0.0015%)]. ^3^ All analytical values are expressed as percentage of food, as fed, unless otherwise indicated. ^4^ Energy was calculated using the modified Atwater factors as described [15]. ^5^ Sum of the SFA: 8:0 + 10:0 + 11:0 + 12:0 + 14:0 + 15:0 + 16:0 + 17:0 + 18:0 + 20:0 + 22:0 + 24:0. ^6^ Sum of the MUFA: 14:1 + 15:1 + 16:1 + 17:1 + 18:1 + 20:1 + 22:1 + 24:1. ^7^ Sum of the PUFA: 18:2 (n-6) + 18:3 (n-6) + 18:3 (n-3) + 18:4 (n-3) + 20:2 (n-6) + 20:3 (n-6) + 20:3 (n-3) + 20:4 (n-6) + 20:4 (n-3) + 20:5 (n-3) + 21:5 (n-3) + 22:2 (n-6) + 22:4 (n-6) + 22:5 (n-6) + 22:5 (n-3) + 22:6 (n-3). ^8^ Sum of the (n-6) fatty acids. ^9^ Sum of the (n-3) fatty acids.

**Table 2:** Mean relative normalized concentrations of selected serum metabolites in cats with different alanine-glyoxylate aminotransferase 2 (AGXT2) single nucleotide polymorphisms (SNPs) from a genome-wide association study (GWAS) of 445 cats in the colony at Hill’s Pet Nutrition, Inc.

	AA	AG	GG
2-oxoarginine	0.45 ± 0.10 ^c^	0.92 ± 0.04 ^b^	1.27 ± 0.04 ^a^
β-aminoisobutyrate	6.53 ± 0.94 ^a^	3.05 ± 0.81 ^b^	1.53 ± 0.81 ^b^
Dimethyl arginines(SDMA + ADMA)	1.26 ± 0.05 ^a^	1.01 ± 0.05 ^b^	1.00 ± 0.05 ^b^

^a,b,c^ Means with different superscripts within a row indicate significant differences between genotypes (*p* < 0.05).

**Table 3:** Body weight and food intake at the end of the 28-day pre-feeding period (Initial) and after consuming control or test food for 28 days.

	Initial	Control Food	Test Food
Body weight (kg)	5.0 ± 0.7	5.0 ± 0.7	5.0 ± 0.8
Food intake (grams)	44.4 ± 17.6	47.4 ± 1.7	45.9 ± 1.7

**Table 4:** Concentrations (mean ± SEM) of selected serum parameters in cats with different alanine-glyoxylate aminotransferase 2 (AGXT2) single nucleotide polymorphisms (SNPs), at the end of the 28-day pre-feeding period (Initial) and after consuming control or test food for 28 days.

Serum Concentrations	AA	AG	GG
Creatinine (mg/dL)			
Initial	1.15 ± 0.06	1.21 ± 0.02	1.26 ± 0.06
Control Food	1.17 ± 0.06	1.18 ± 0.09	1.21 ± 0.06
Test Food	1.17 ± 0.06	1.15 ± 0.09	1.16 ± 0.06
Urea (mg/dL)			
Initial	23.3 ± 1.3	19.9 ± 0.8	22.2 ± 1.5
Control Food	23.3 ± 1.0	19.9 ± 1.5	21.4 ± 1.0
Test Food	22.0 ± 1.0	20.8 ± 1.5	21.8 ± 1.0
Albumin (mg/dL)			
Initial	3.20 ± 0.07	3.50 ± 0.13	3.58 ± 0.10
Control Food	3.38 ± 0.10	3.45 ± 0.14	3.59 ± 0.10
Test Food	3.25 ± 0.10	3.45 ± 0.14	3.46 ± 0.10
Protein (mg/dL)			
Initial	6.77 ± 0.17	7.00 ± 0.25	6.84 ± 0.18
Control Food	7.14 ± 0.21	7.09 ± 0.30	7.01 ± 0.20
Test Food	7.03 ± 0.21	6.87 ± 0.30	6.99 ± 0.20
Cholesterol (mg/dL)			
Initial	142 ± 16	172 ± 17	152 ± 18
Control Food	152 ± 13	188 ± 18	173 ± 13
Test Food	155 ± 13	200 ± 18	181 ± 13
Triglycerides (mg/dL)			
Initial	32.8 ± 2.4	45.5 ± 10.0	37.6 ± 4.2
Control Food	36.7 ^a^ ± 3.6	43.3 ± 10.0	32.8 ± 4.2
Test Food	30.8 ^b,d^ ± 3.6	46.1 ^c^ ± 10.0	32.3 ^d^ ± 4.2
Calcium (mg/dL)			
Initial	9.44 ± 0.14	9.82 ± 0.17	9.83 ± 0.12
Control Food	9.57 ± 0.13	9.65 ± 0.19	9.71 ± 0.12
Test Food	9.54 ± 0.13	9.57 ± 0.19	9.60 ± 0.12

^a,b^ Means with different superscripts within a column indicate significant pairwise differences (*p* < 0.05). ^c,d^ Means with different superscripts within a row indicate significant differences between genotypes (*p* < 0.05).

**Table 5:** Concentrations (natural log of means ± SEM) of selected serum cytokines in cats with different alanine-glyoxylate aminotransferase 2 (AGXT2) single nucleotide polymorphisms (SNPs), averaged across time (there was no effect of food and no interaction of food and time).

Serum Concentrations	AA	AG	GG
Interleuken 1-β (ln pg/mL)	2.21 ± 0.27 ^b^	2.95 ± 0.41 ^a,b^	3.10 ± 0.17 ^a^
Interleuken 2 (ln pg/mL)	1.87 ± 0.35 ^a,b^	0.88 ± 0.53 ^b^	2.81 ± 0.34 ^a^
Interleuken 4 (ln pg/mL)	4.73 ± 0.73 ^b^	6.22 ± 0.51 ^a^	5.47 ± 0.33 ^a,b^
Interleuken 6 (ln pg/mL)	3.56 ± 0.26 ^b^	4.63 ± 0.40 ^a^	4.52 ± 0.26 ^a^
Interleuken 8 (ln pg/mL)	2.73 ± 0.18	3.23 ± 0.16	3.22 ± 0.17
Interleuken 18 (ln pg/mL)	4.57 ± 0.28 ^a,b^	3.62 ± 0.42 ^b^	4.97 ± 0.28 ^a^
MCP-1 (ln pg/mL)	6.91 ± 0.21	7.40 ± 0.32	7.23 ± 0.21
Interleuken 13 (ln pg/mL)	2.02 ± 0.27 ^b^	1.69 ± 0.41 ^b^	2.70 ± 0.17 ^a^
Flt 3L (ln pg/mL)	3.90 ± 0.17	3.98 ± 0.25	3.83 ± 0.16
TNF-α (ln pg/mL)	0.93 ± 0.47 ^b^	2.89 ± 0.70 ^a^	2.62 ± 0.46 ^a^
KC ^1^ (ln pg/mL)	-0.91 ± 0.40 ^b^	-0.62 ± 0.61 ^a,b^	0.53 ± 0.18 ^a^
RANTES ^2^ (ln pg/mL)	2.53 ± 0.17 ^b^	3.04 ± 0.2,6 ^a,b^	3.16 ± 0.17 ^a^
Interferon-γ (ln pg/mL)	3.09 ± 0.37 ^b^	3.86 ± 0.56 ^a,b^	4.13 ± 0.37 ^a^
Stem cell factor (ln pg/mL)	4.67 ± 0.12 ^b^	5.39 ± 0.18 ^a^	5.14 ± 0.12 ^a^
Interleuken 12 p40 (ln pg/mL)	5.62 ± 0.21	5.77 ± 0.31	5.65 ± 0.20
PDGF ^3^ BB (ln pg/mL)	4.05 ± 0.37	3.10 ± 0.55	4.47 ± 0.36
SDF-1 ^4^ (ln pg/mL)	5.61 ± 0.34	6.64 ± 0.52	6.04 ± 0.34
FAS ^5^ (ln pg/mL)	3.57 ± 0.26 ^b^	4.63 ± 0.40 ^a^	4.52 ± 0.26 ^a^

^1^ Keratinocyte chemoattractant. ^2^ Regulated upon Activation, Normal T cell Expressed and Secreted; chemokine ligand, also known as CCL5. ^3^ Platelet-derived growth factor. ^4^ Stromal cell derived factor–1. ^5^ Fas is FS7-associated cell surface antigen; also known a CD95, APO-1 or TNFRSF6. ^a,b^ Means with different superscripts within a row indicate significant differences between genotypes (*p* < 0.05).

**Table 6:** Values (mean ± SEM) for selected urine parameters in cats with different alanine-glyoxylate aminotransferase 2 (AGXT2) single nucleotide polymorphisms (SNPs), at the end of the 28-day pre-feeding period (Initial) and after consuming control or test food for 28 days.

Urine Parameters	AA	AG	GG
Calcium (mg/dL)			
Initial	3.10 ± 1.27	3.22 ± 1.58	3.36 ± 0.99
Control Food	3.42 ± 0.39	3.16 ± 0.56	3.72 ± 0.38
Test Food	3.78 ± 0.39	3.13 ± 0.56	3.06 ± 0.39
Creatinine (mg/dL)			
Initial	329 ± 80	414 ± 31	447 ± 74
Control Food	357 ± 27	388 ± 38	438 ± 28
Test Food	346 ± 27	369 ± 38	397 ± 27
Protein to Creatinine ratio			
Initial	0.16 ± 0.02	0.14 ± 0.03	0.16 ± 0.02
Control Food	0.17 ± 0.02	0.15 ± 0.03	0.16 ± 0.02
Test Food	0.15 ± 0.02	0.13 ± 0.03	0.15± 0.02
Oxalate (µM)			
Initial	NA	NA	NA
Control Food	1042 ± 101	1337 ± 143	1011 ± 95
Test Food	822 ± 101	877 ± 143	910 ± 94
pH			
Initial	6.30 ± 0.12	6.30 ± 0.25	6.31 ± 0.09
Control Food	6.11 ± 0.14	5.93 ± 0.19	6.35 ± 0.13
Test Food	6.18 ± 0.14	6.16 ± 0.19	6.41 ± 0.13
Specific gravity			
Initial	1.044 ± 0.011	1.045 ± 0.001	1.050 ± 0.003
Control Food	1.048 ± 0.003	1.049 ± 0.004	1.049 ± 0.002
Test Food	1.043 ± 0.003	1.046 ± 0.004	1.049 ± 0.002
Fractional excretion of calcium (%)			
Initial	0.115 ± 0.046	0.091 ± 0.047	0.094 ± 0.038
Control Food	0.119 ± 0.017	0.100 ± 0.024	0.103 ± 0.018
Test Food	0.139 ± 0.017	0.102 ± 0.024	0.091 ± 0.017
Struvite relative super saturation (ln)			
Initial	−1.42 ± 0.40	−0.46 ± 0.36	−0.27 ± 0.40
Control Food	−0.97 ± 0.38	−1.03 ± 0.54	−0.04 ± 0.38
Test Food	−1.10 ± 0.38	−0.96 ± 0.54	0.06 ± 0.38
Calcium oxalate titration test (ln 1/L)			
Initial	2.55 ± 0.25	2.15 ± 0.61	2.58 ± 0.21
Control Food	2.67 ± 0.29	2.56 ± 0.40	2.74 ± 0.28 ^a^
Test Food	2.94 ± 0.29 ^c^	2.18 ± 0.40 ^c,d^	2.12 ± 0.28 ^b,d^

^a,b^ Means with different superscripts within a column indicate significant pairwise differences (*p* < 0.05). ^c,d^ Means with different superscripts within a row indicate significant differences between genotypes (*p* < 0.05). NA = not available.

**Table 7:** Ratios of the concentrations of selected serum metabolites in cats with different alanine-glyoxylate aminotransferase 2 (AGXT2) single nucleotide polymorphisms (SNPs) that were significantly different (all analytes that had a *p* ≤ 0.05 and a *q* ≤0.1 for an effect of food) after consuming control or test food for 28 days. Green denotes a decline and red an increase in the ratio (test food concentration/control food concentration) of each metabolite.

Metabolism Pathway	Biochemical Name	AA Test/Control	AG Test/Control	GG Test/Control
Glycine, Serine, Threonine Metabolism	sarcosine	1.39	1.76	1.64
	betaine	2.06	2.6	2.28
	N-acetylthreonine	0.85	1.08	0.85
Alanine and Asparagine Metabolism	N-methylalanine	1.18	1.58	2.51
	N,N-dimethylalanine	0.51	0.48	0.45
	N-acetylasparagine	0.85	1.02	0.81
	Hydroxyasparagine	0.85	0.96	0.81
Glutamine Metabolism	glutamine	0.95	1.05	0.95
	α-ketoglutaramate	1.2	1.67	1.26
	Pyroglutamine	1.3	1.83	1.28
	N-acetyl-aspartyl-glutamate (NAAG)	0.89	1.42	0.9
Histidine Metabolism	imidazole propionate	1.21	1.88	1.29
	carnosine	0.94	1.2	0.91
	N-acetylcarnosine	0.91	1.2	0.9
Lysine Metabolism	N6,N6-dimethyllysine	0.95	1.15	0.94
	N6,N6,N6-trimethyllysine	0.95	1.13	0.91
	hydroxy-N6,N6,N6-trimethyllysine	0.79	1.29	0.7
	5-(galactosylhydroxy)-L-lysine	0.88	1.7	0.73
	glutarylcarnitine (C5-DC)	0.83	1.15	0.71
Tryptophan Metabolism	C-glycosyltryptophan	0.84	1.24	0.89
Leucine, Isoleucine, Valine Metabolism	α-hydroxyisocaproate	0.88	0.96	0.88
	3-methylglutaconate	0.96	1.22	0.88
	2-methylbutyrylcarnitine (C5)	1.04	1.4	0.8
	tiglylcarnitine (C5:1-DC)	0.8	0.88	0.8
	α-hydroxyisovalerate	0.91	1.09	0.85
	isobutyrylcarnitine (C4)	0.95	1.17	0.82
Methionine, Cysteine, Metabolism	methionine	1.07	1.26	1.12
	N-acetylmethionine	0.83	1.16	0.87
	N-formylmethionine	0.91	1.17	0.89
	methionine sulfoxide	1.06	1.24	1.17
Proline Metabolism	trans-4-hydroxyproline	0.68	0.63	0.65
	N-methylproline	0.93	0.97	0.72
Creatine Metabolism	creatinine	0.98	1.05	0.95
Polyamine Metabolism	N-acetyl-isoputreanine	0.92	1.11	0.91
Glutathione Metabolism	2-hydroxybutyrate/2-hydroxyisobutyrate	0.84	0.86	0.8
γ-glutamyl Amino Acid Metabolism	γ-glutamyl-epsilon-lysine	0.9	1.19	0.76
Dipeptide Metabolism	leucylhydroxyproline	1.27	1.52	1.68
Energy Metabolism	malate	0.84	0.98	0.87
Fatty Acid Metabolism	malonylcarnitine	0.9	0.89	0.58
	malonate	1.51	1.2	0.85
	heptanoate (7:0)	0.84	0.91	0.75
Long Chain Polyunsaturated Fatty Acid (n3 and n6) Metabolism	tetradecadienoate (14:2)	0.92	0.94	0.74
	hexadecatrienoate (16:3n3)	0.78	1.06	0.86
	(11 or 12)-methyltridecanoate (a14:0 or i14:0)	0.8	0.91	0.9
Fatty Acid, Dicarboxylate	glutarate (C5-DC)	0.92	0.78	0.81
	dodecadienoate (12:2)	0.96	1	0.76
	branched chain 14:0 dicarboxylic acid	0.95	1.65	0.95
	hexadecanedioate (C16-DC)	0.75	1.22	0.85
	hexadecenedioate (C16:1-DC)	0.83	1.38	0.96
	heptadecanedioate (C17-DC)	0.74	1.24	0.82
	octadecanedioate (C18-DC)	0.83	1.27	0.84
	nonadecanedioate (C19-DC)	0.88	1.32	0.79
	eicosanedioate (C20-DC)	0.89	1.36	0.84
	docosadioate (C22-DC)	0.75	1.35	0.83
	butyrylglycine	0.66	0.79	0.66
Fatty Acid Metabolism (Acyl Glycine)	hexanoylglycine	0.67	0.66	0.53
	3,4-methylene heptanoylglycine	0.7	0.55	0.76
	N-octanoylglycine	0.65	0.92	0.54
	lignoceroylcarnitine (C24)	0.82	0.89	0.81
	cerotoylcarnitine (C26)	0.8	0.91	0.62
Fatty Acid Acyl Carnitine Metabolism	nervonoylcarnitine (C24:1)	0.82	1.29	0.75
	ximenoylcarnitine (C26:1)	0.72	0.76	0.61
	adrenoylcarnitine (C22:4)	0.78	1.3	0.72
	docosahexaenoylcarnitine (C22:6)	0.93	1.44	0.87
Fatty Acyl Carnitine dicarboxylate Metabolism	adipoylcarnitine (C6-DC)	0.8	1.05	0.81
	suberoylcarnitine (C8-DC)	0.68	0.52	0.69
Fatty Acid Acyl Carnitine hydroxy Metabolism	(R)-3-hydroxybutyrylcarnitine	1.03	1.68	0.38
	3-hydroxyoleoylcarnitine	0.89	1.23	0.81
Fatty Acid Mono hydroxyl Metabolism	2-hydroxyoctanoate	0.87	1.13	0.72
	2-hydroxylaurate	0.85	1.07	0.83
	3-hydroxyoctanoate	1.11	0.86	0.75
	3-hydroxydecanoate	1	0.99	0.78
	3-hydroxysebacate	0.89	1	0.76
	16-hydroxypalmitate	0.85	1.12	0.85
Endocannabinoid Metabolism	hexanoyltaurine	0.83	1.14	0.75
Lysophospholipid Metabolism	1-linoleoyl-GPA (18:2)	0.78	0.87	0.67
	1-palmitoyl-GPE (16:0)	0.84	1.26	0.9
	1-stearoyl-GPE (18:0)	0.86	1.4	0.91
	1-arachidonoyl-GPE (20:4n6)	0.83	1.41	0.96
	1-(1-enyl-stearoyl)-2-oleoyl-GPE (P-18:0/18:1)	0.76	1.13	0.95
Diacylglycerol Metabolism	oleoyl-linoleoyl-glycerol (18:1/18:2)	0.63	1.52	0.93
Mevalonate Metabolism	3-hydroxy-3-methylglutarate	0.93	1.13	0.92
Pyrimidine Metabolism	3-ureidopropionate	0.95	1.06	0.93
	3-(3-amino-3-carboxypropyl) uridine	0.79	1.17	0.78
	5-methyl-2’-deoxycytidine	1	1.2	1.15
	nicotinamide	0.89	1.19	0.9
	gulonate	0.77	1.1	0.92
	4-allylcatechol sulfate	2.86	2.92	3.58
Xanthine Metabolism	theophylline	44.08	42.57	35.37
Food Component	ergothioneine	1.18	1.49	1.25
	methyl indole-3-acetate	0.8	2.07	0.9
	eugenol sulfate	28.82	21.9	33.99
	(2,4 or 2,5)-dimethylphenol sulfate	0.71	0.73	0.6
Chemical	sulfate	0.9	1.05	0.93
	1,2,3-benzenetriol sulfate	2.23	15.29	11.31
Partially Characterized Molecules	glycine conjugate of C10H14O2	0.72	0.89	0.8

**Table 8:** Scaled imputed values of the concentrations of selected genotype-specific serum metabolites in cats with different alanine-glyoxylate aminotransferase 2 (AGXT2) single nucleotide polymorphisms (SNPs) after consuming control or test food for 28 days.

Serum Concentrations	AA	AG	GG
2-oxoarginine ^1^			
Initial	0.65 ± 0.30	1.82 ± 0.45	2.21 ± 0.28
Control Food	0.78 ± 0.30	1.79 ± 0.46	2.12 ± 0.29
Test Food	0.76 ± 0.30	2.10 ± 0.46	2.11 ± 0.29
β-aminoisobutyrate ^1^			
Initial	1.67 ± 0.28	0.57 ± 0.42	0.63 ± 0.26
Control Food	1.78 ± 0.29	0.55 ± 0.44	0.60 ± 0.28
Test Food	2.21 ± 0.29	0.62 ± 0.44	0.78 ± 0.28
Oxalate			
Initial	1.06 ± 0.12	1.13 ± 0.18	1.11 ± 0.11
Control Food	1.01 ± 0.11	1.02 ± 0.18	1.04 ± 0.11
Test Food	0.94 ± 0.12	1.01 ± 0.18	0.95 ± 0.11
Dimethyl arginines (SDMA + ADMA)			
Initial	1.07 ± 0.06	0.96 ± 0.10	0.96 ± 0.06
Control Food	1.11 ± 0.06	0.94 ± 0.08	0.99 ± 0.05
Test Food	1.06 ± 0.06	1.02 ± 0.08	0.94 ± 0.05
S-adenosylhomocysteine ^1^			
Initial	1.01 ± 0.08	0.93 ± 0.13	1.11 ± 0.08
Control Food	0.97 ± 0.17	0.70 ± 0.25 ^b^	1.19 ± 0.16 ^b^
Test Food	1.04 ± 0.17	0.99 ± 0.25 ^a^	1.75 ± 0.16 ^a^
Oxidized glutathione ^2^			
Initial	0.84 ± 0.12	0.99 ± 0.19	1.18 ± 0.12
Control Food	0.97 ± 0.18	0.91 ± 0.27	1.45 ± 0.17 ^a^
Test Food	0.95 ± 0.18	1.25 ± 0.27	1.13 ± 0.17 ^b^
Cortisol ^1^			
Initial	0.97 ± 0.30	1.37 ± 0.44	1.69 ± 0.28
Control Food	0.94 ± 0.26	1.13 ± 0.39	1.80 ± 0.24
Test Food	0.74 ± 0.26	0.85 ± 0.39	1.27 ± 0.24
9-HODE + 13-HODE ^1^			
Initial	1.60 ± 0.16	1.01 ± 0.24	1.13 ± 0.15
Control Food	1.56 ± 0.14	1.02 ± 0.21	1.02 ± 0.13
Test Food	1.20 ± 0.14	0.73 ± 0.21	0.84 ± 0.13

^1^ Significant difference between genotypes (*p* < 0.05). ^2^ Significant genotypes × food interaction (*p* < 0.05). ^a,b^ Means with different superscripts within a column indicate significant pairwise differences (*p* < 0.05).

## Data Availability

All relevant data are within the paper and its Supplementary Information files.

## Supplementary Materials

The following are available online at https://www.mdpi.com/article/10.3390/genes13050791/s1. Table S1: Heat map of serum biochemicals from cats profiled in this study. Red and green shaded cells indicate *p* ≤ 0.05 (red indicates that the mean values are significantly higher for that comparison; green values significantly lower). Light red and light green shaded cells indicate 0.05 < *p*< 0.10 (light red indicates that the mean values trend higher for that comparison; light green values trend lower).

## References

[1] Ordovas J.M., Ferguson L.R., Tai E.S., Mathers J.C. (2018). Personalised nutrition and health. BMJ.

[2] Peneş N.O., Weber B., Păun S.D. (2017). Role of genetic polymorphism in nutritional supplementation therapy in personalized medicine. Rom. J. Morphol. Embryol..

[3] Bush C.L., Blumberg J.B., El-Sohemy A., Minich D.M., Ordovás J.M., Reed D.G., Behm V.A.Y. (2019). Toward the Definition of Personalized Nutrition: A Proposal by The American Nutrition Association. J. Am. Coll. Nutr..

[4] Barreiro L.B., Laval G., Quach H., Patin E., Quintana-Murci L. (2008). Natural selection has driven population differentiation in modern humans. Nat. Genet..

[5] Hall J.A., Brockman J.A., Davidson S.J., MacLeay J.M., Jewell D.E. (2017). Increased dietary long-chain polyunsaturated fatty acids alter serum fatty acid concentrations and lower risk of urine stone formation in cats. PLoS ONE.

[6] Lulich J.P., Kruger J.M., MacLeay J.M., Merrills J.M., Paetau-Robinson I., Albasan H., Osborne C.A. (2013). Efficacy of two commercially available, low-magnesium, urine-acidifying dry foods for the dissolution of struvite uroliths in cats. J. Am. Veter Med. Assoc..

[7] Salido E., Pey A.L., Rodriguez R., Lorenzo V. (2012). Primary hyperoxalurias: Disorders of glyoxylate detoxification. Biochim. Biophys. Acta Mol. Basis Dis..

[8] Cochat P., Rumsby G. (2013). Primary Hyperoxaluria. N. Engl. J. Med..

[9] Hu X.-L., Li M.-P., Song P.-Y., Tang J., Chen X.-P. (2017). AGXT2: An unnegligible aminotransferase in cardiovascular and urinary systems. J. Mol. Cell. Cardiol..

[10] Kittel A., Müller F., Koenig J., Mieth M., Sticht H., Zolk O., Kralj A., Heinrich M., Fromm M.F., Maas R. (2014). Alanine-glyoxylate aminotransferase 2 (AGXT2) Polymorphisms Have Considerable Impact on Methylarginine and beta-aminoisobutyrate Metabolism in Healthy Volunteers. PLoS ONE.

[11] Jewell D.E., Panickar K.S. (2021). Botanicals Reduce Circulating Concentrations of Cholesterol and Triglycerides and Work Synergistically with Arachidonic Acid to Reduce Inflammatory Cytokines in Cats. Front. Veter Sci..

[12] NRC (2011). Guide for the Care and Use of Laboratory Animals.

[13] Chang C.C., Chow C.C., Tellier L.C., Vattikuti S., Purcell S.M., Lee J.J. (2015). Second-generation PLINK: Rising to the challenge of larger and richer datasets. Gigascience.

[14] Purcell S., Neale B., Todd-Brown K., Thomas L., Ferreira M.A.R., Bender D., Maller J., Sklar P., de Bakker P.I.W., Daly M.J. (2007). PLINK: A Tool Set for Whole-Genome Association and Population-Based Linkage Analyses. Am. J. Hum. Genet..

[15] Hall J.A., Melendez L.D., Jewell D.E. (2013). Using Gross Energy Improves Metabolizable Energy Predictive Equations for Pet Foods Whereas Undigested Protein and Fiber Content Predict Stool Quality. PLoS ONE.

[16] Hall J., Yerramilli M., Obare E., Jewell D. (2014). Comparison of Serum Concentrations of Symmetric Dimethylarginine and Creatinine as Kidney Function Biomarkers in Cats with Chronic Kidney Disease. J. Veter Intern. Med..

[17] Hesse A., Bongartz D., Heynck H., Berg W. (1996). Measurement of urinary oxalic acid: A comparison of five methods. Clin. Biochem..

[18] Werness P.G., Brown C.M., Smith L.H., Finlayson B. (1985). Equil2: A Basic Computer Program for the Calculation of Urinary Saturation. J. Urol..

[19] Robert M., Boularan A.M., Colette C., Averous M., Monnier M. (1994). Urinary calcium oxalate saturation in ‘stone formers’ and normal subjects: An application of the EQUIL2program. Br. J. Urol..

[20] Milošević D., Batinić D., Blau N., Konjevoda P., Štambuk N., Votava-Raić A., Barbarić V., Fumić K., Rumenjak V., Stavljenić-Rukavina A. (1998). Determination of urine saturation with computer program EQUIL 2 as a method for estimation of the risk of urolithiasis. J. Chem. Inf. Comput. Sci..

[21] Laube N., Labedzke V., Hergarten S., Hesse A. (2002). Determination of Urinary Calcium-Oxalate Formation Risk with BONN-Risk-Index and EQUIL Applied to a Family. J. Chem. Inf. Comput. Sci..

[22] Laube N., Schneider A., Hesse A. (2000). A new approach to calculate the risk of calcium oxalate crystallization from unprepared native urine. Urol. Res..

[23] Kavanagh J.P., Laube N. (2006). Why Does the Bonn Risk Index Discriminate Between Calcium Oxalate Stone Formers and Healthy Controls?. J. Urol..

[24] Hall J.A., Jackson M., Vondran J.C., Vanchina M.A., Jewell D.E. (2018). Comparison of circulating metabolite concentrations in dogs and cats when allowed to freely choose macronutrient intake. Biol. Open.

[25] Hu X.-L., Zeng W.-J., Li M.-P., Yang Y.-L., Kuang D.-B., Li H., Zhang Y.-J., Jiang C., Peng L.-M., Qi H. (2017). AGXT2 rs37369 polymorphism predicts the renal function in patients with chronic heart failure. Gene.

[26] Jarzebska N., Georgi S., Jabs N., Brilloff S., Maas R., Rodionov R.N., Zietz C., Montresor S., Hohenstein B., Weiss N. (2019). Kidney and liver are the main organs of expression of a key metabolic enzyme alanine:glyoxylate aminotransferase 2 in humans. Atheroscler. Suppl..

[27] Rodionov R.N., Jarzebska N., Weiss N., Lentz S.R. (2014). AGXT2: A promiscuous aminotransferase. Trends Pharmacol. Sci..

[28] Suhre K., Wallaschofski H., Raffler J., Friedrich N., Haring R., Michael K., Wasner C., Krebs A., Kronenberg F., Chang D. (2011). A genome-wide association study of metabolic traits in human urine. Nat. Genet..

[29] Rhee E.P., Ho J., Chen M.-H., Shen D., Cheng S., Larson M., Ghorbani A., Shi X., Helenius I.T., O’Donnell C.J. (2013). A Genome-wide Association Study of the Human Metabolome in a Community-Based Cohort. Cell Metab..

[30] Caplin B., Wang Z., Slaviero A., Tomlinson J., Dowsett L., Delahaye M., Salama A., Wheeler D.C., Leiper J., The International Consortium for Blood Pressure Genome-Wide Association Studies (2012). Alanine-Glyoxylate Aminotransferase-2 Metabolizes Endogenous Methylarginines, Regulates NO, and Controls Blood Pressure. Arter. Thromb. Vasc. Biol..

[31] Kittel A., Maas R., König J., Mieth M., Weiss N., Jarzebska N., Hohenstein B., Martens-Lobenhoffer J., Bode-Böger S.M., Rodionov R.N. (2013). In vivo evidence that Agxt2 can regulate plasma levels of dimethylarginines in mice. Biochem. Biophys. Res. Commun..

[32] Turner M.A., Yang X., Yin D., Kuczera K., Borchardt R.T., Howell P.L. (2000). Structure and Function of S-Adenosylhomocysteine Hydrolase. Cell Biophys..

[33] Papasotiriou M., Genovese F., Klinkhammer B.M., Kunter U., Nielsen S.H., Karsdal M.A., Floege J., Boor P. (2015). Serum and urine markers of collagen degradation reflect renal fibrosis in experimental kidney diseases. Nephrol. Dial. Transplant..

[34] Vangaveti V., Baune B.T., Kennedy R.L. (2010). Hydroxyoctadecadienoic acids: Novel regulators of macrophage differentiation and atherogenesis. Ther. Adv. Endocrinol. Metab..

[35] Wolkow A., Aisbett B., Reynolds J., Ferguson S.A., Main L.C. (2015). Relationships between inflammatory cytokine and cortisol responses in firefighters exposed to simulated wildfire suppression work and sleep restriction. Physiol. Rep..

[36] Jia Y., Liu L., Sheng C., Cheng Z., Cui L., Li M., Zhao Y., Shi T., Yau T.O., Li F. (2019). Increased Serum Levels of Cortisol and Inflammatory Cytokines in People with Depression. J. Nerv. Ment. Dis..

