# Supplementary material for: Cats with Genetic Variants of AGXT2 Respond Differently to a Dietary Intervention Known to Reduce the Risk of Calcium Oxalate Stone Formation
Source: Genes (Basel). 2022 Apr 28;13(5):791. doi: 10.3390/genes13050791 (PMC9141165; doi:10.3390/genes13050791)
Supplement: Supplementary file 1 [file genes-13-00791-s001.zip › Table S1.pdf]

Table S1. All untargeted biochemicals which had a p<0.1 when concentration on test food was compared to control in any genotype.

Biochemical Name	GG TEST / GG CTRL	AA TEST / AA CTRL	AG TEST / AG CTRL	GG TEST / GG CTRL p-value	GG TEST / GG CTRL q-value	AA TEST / AA CTRL p-value	AA TEST / AA CTRL q-value	AG TEST / AG CTRL p-value	AG TEST / AG CTRL q-value
glycine	0.92	0.97	1.03	0.029960069	0.135062123	0.660726337	0.504794224	0.840400425	0.576407059
sarcosine	1.64	1.39	1.76	0.000817492	0.036734416	0.002966577	0.075164263	0.000245183	0.041088109
dimethylglycine	1.14	1.09	1.27	0.254062933	0.407229788	0.620218065	0.49294256	0.036087896	0.162467472
betaine	2.28	2.06	2.6	2.072E-08	3.9852E-06	1.55614E-06	0.00024971	0.000125404	0.031522945
N-acetylserine	0.93	0.9	1.17	0.022178431	0.120570985	0.096839527	0.257857023	0.007357053	0.127932774
N-acetylthreonine	0.85	0.85	1.08	0.003008041	0.047614135	0.042590858	0.204306918	0.165227541	0.243589669
allo-threonine	1.11	0.54	0.91	0.665594632	0.625988494	0.008650362	0.122479891	0.854860249	0.579804408
N-methylalanine	2.51	1.18	1.58	0.00100785	0.036734416	0.459420935	0.431678512	0.144004028	0.226280918
N,N-dimethylalanine	0.45	0.51	0.48	0.026829143	0.127361579	1.3344E-05	0.001284769	0.11517405	0.210556643
aspartate	0.89	0.91	1.17	0.107951669	0.252450363	0.093771429	0.256488137	0.274487383	0.313629361
asparagine	0.92	0.95	1.06	0.026467446	0.127361579	0.078668808	0.245916263	0.277790611	0.315048798
N-acetylasparagine	0.81	0.85	1.02	0.066491851	0.196758971	0.002238858	0.066390469	0.850840387	0.579613827
hydroxyasparagine	0.81	0.85	0.96	0.001171973	0.036734416	0.007197362	0.115494559	0.148930524	0.229674679
glutamate	0.91	0.93	1.22	0.051068426	0.171326585	0.161248381	0.318008092	0.077800464	0.190803079
glutamine	0.95	0.95	1.05	0.013732544	0.097577835	0.167343068	0.323581916	0.258428132	0.304984095
alpha-ketoglutaramate	1.26	1.2	1.67	0.002551594	0.047614135	0.070742044	0.235121437	0.006618531	0.127932774
pyroglutamine	1.28	1.3	1.83	0.006079745	0.068150556	0.001831886	0.066390469	0.009644319	0.134690715
N-acetyl-aspartyl-glutamate (NAAG)	0.9	0.89	1.42	0.046464832	0.164489242	0.004672102	0.094447584	0.047530503	0.182661208
2-pyrrolidinone	0.83	0.92	1.24	0.033347496	0.138620355	0.182845047	0.332913203	0.288895191	0.321717856
histidine	1.01	0.99	1.15	0.705123226	0.632540422	0.724474833	0.519117628	0.005699254	0.127932774
3-methylhistidine	0.97	0.96	1.06	0.676910739	0.625988494	0.423685625	0.425811742	0.071855584	0.188745601
N-acetylhistidine	0.92	0.85	0.81	0.697645821	0.630604564	0.407458987	0.420351028	0.045598932	0.182661208
imidazole propionate	1.29	1.21	1.88	0.004505775	0.061660247	0.068096221	0.235121437	0.003626925	0.110350299
formiminoglutamate	1.11	0.97	1.47	0.240347511	0.396286516	0.77552359	0.533096551	0.014092635	0.147700418

carnosine	**0.91**	0.94	**1.2**	0.025251276	0.127361579	0.104627845	0.265076514	0.000742393	0.067440674
N-acetylcarnosine	0.9	**0.91**	**1.2**	0.10681301	0.252450363	0.014342031	0.153815112	0.001529669	0.085447992
anserine	0.78	0.87	**1.51**	0.127876409	0.273019429	0.124122865	0.280279855	0.029008873	0.158522177
histamine	0.96	1.14	1.65	0.949208365	0.690699436	0.46090788	0.431678512	0.204897736	0.267561383
1-methyl-5-imidazoleacetate	1.01	1.11	**1.47**	0.844658453	0.667630561	0.570838732	0.477937813	0.023455001	0.157654649
1-ribosyl-imidazoleacetate	1.11	**0.89**	**1.35**	0.415205689	0.521363947	0.038578467	0.204306918	0.051851087	0.185067015
4-imidazoleacetate	**1.43**	1.2	**1.85**	0.018004197	0.11002535	0.203167304	0.343621116	0.050573966	0.185067015
lysine	1	1.02	1.1	0.780769641	0.658574968	0.583527271	0.481014012	0.171082479	0.243589669
N6-acetyllysine	0.95	0.96	**1.32**	0.37934374	0.49914771	0.682990199	0.510478406	0.021773323	0.157654649
N6-methyllysine	**0.95**	0.99	0.99	0.065587108	0.195082128	0.348136766	0.402681864	0.99362639	0.613068922
N6,N6-dimethyllysine	**0.94**	0.95	**1.15**	0.006141916	0.068155556	0.28210927	0.368909448	0.006594369	0.127932774
N6,N6,N6-trimethyllysine	**0.91**	0.95	**1.13**	0.004701725	0.061660247	0.502786117	0.446573973	0.093073198	0.20282035
hydroxy-N6,N6,N6-trimethyllysine	**0.7**	0.79	1.29	0.001209556	0.036734416	0.139390015	0.295607347	0.23514118	0.288331332
5-(galactosylhydroxy)-L-lysine	**0.73**	0.88	1.7	0.001051721	0.036734416	0.334492492	0.396615568	0.181360472	0.248242476
2-oxoadipate	1.03	**0.78**	0.91	0.983322618	0.699724318	0.043171484	0.204306918	0.522379539	0.439532946
glutarylcarnitine (C5-DC)	**0.71**	0.83	1.15	0.00093035	0.036734416	0.109527578	0.267649647	0.349466829	0.36092854
6-oxopiperidine-2-carboxylate	**0.87**	**0.85**	**1.23**	0.015245115	0.101114206	0.043249665	0.204306918	0.044032607	0.181451923
5-aminovalerate	**1.28**	1	1.22	0.029594687	0.134465465	0.83025328	0.546767435	0.348042746	0.36092854
N,N,N-trimethyl-5-aminovalerate	0.9	1	**1.37**	0.227771571	0.389532061	0.676122308	0.506990305	0.035164008	0.162467472
phenylalanine	**0.95**	**0.95**	1.09	0.029577313	0.134465465	0.056330593	0.223882146	0.129386944	0.220503478
N-acetylphenylalanine	0.87	**0.83**	1.14	0.175341135	0.333919604	0.005620234	0.102362942	0.385655129	0.379424363
phenyllactate (PLA)	0.99	**0.88**	1.09	0.965569561	0.695684408	0.085069734	0.24917401	0.593739191	0.469977996
2-hydroxyphenylacetate	**1.09**	0.96	1.08	0.086488483	0.22684872	0.456646516	0.431614187	0.202984348	0.267324439
tyrosine	0.98	0.92	**1.15**	0.375447209	0.497276584	0.247474319	0.355735911	0.085820341	0.198827981
N-acetyltyrosine	**0.81**	**0.85**	1.22	0.017389436	0.109068265	0.082633391	0.247203017	0.174559076	0.244267837
m-tyramine sulfate	1.01	**0.81**	1.07	0.740079089	0.643147796	0.040843454	0.204306918	0.478527312	0.423550693
3-(4-hydroxyphenyl)lactate	0.97	0.91	**1.15**	0.229600969	0.390138436	0.131152652	0.283120417	0.068147978	0.188745601

dopamine 3-O-sulfate	0.91	0.87	**1.36**	0.139117876	0.288761225	0.671459025	0.505828125	0.016586722	0.147700418
3-hydroxyphenylacetate sulfate	3.03	**1.58**	3.01	0.239174495	0.396286516	0.053260376	0.217285946	0.473268021	0.421176529
C-glycosyltryptophan	**0.89**	**0.84**	**1.24**	0.006690699	0.070195544	0.037433714	0.204306918	0.04531646	0.182661208
kynurenine	0.99	0.93	**1.2**	0.731979849	0.639316219	0.199809726	0.34325149	0.055127762	0.187851722
anthranilate	**1.73**	0.79	0.92	0.06354534	0.192184883	0.32969673	0.391894378	0.625002286	0.482836437
5-hydroxyindoleacetate	1.03	0.85	**1.55**	0.531349985	0.560523826	0.42264692	0.425811742	0.015563332	0.147700418
indolelactate	0.96	**0.9**	1.14	0.416267497	0.521363947	0.042912584	0.204306918	0.291012708	0.322531196
indoleacetylglycine	**0.75**	0.84	1.17	0.087380067	0.227122484	0.142206471	0.29764661	0.328173115	0.345884915
indoleacetoylcarnitine	1.58	**1.59**	**3.88**	0.141245234	0.29212608	0.099763583	0.259603011	0.017999153	0.149327958
5-hydroxyindole sulfate	0.68	**0.62**	2.17	0.438910316	0.527996766	0.080406119	0.246546513	0.197155563	0.262914591
3-indoxyl sulfate	0.85	0.76	**1.5**	0.90948999	0.681541341	0.860762315	0.553236525	0.017711234	0.149327958
6-bromotryptophan	**0.9**	**0.87**	1.1	0.068973741	0.199000661	0.068801597	0.235121437	0.153064544	0.233247518
1-carboxyethylleucine	0.89	**0.81**	1.08	0.21139935	0.370773325	0.058597703	0.223882146	0.787675723	0.558532215
alpha-hydroxyisocaproate	**0.88**	0.88	0.96	0.009720701	0.091953548	0.228981997	0.352181402	0.759875659	0.546156183
isovalerylglycine	0.94	**0.75**	0.96	0.324965684	0.454701605	0.027781155	0.201009166	0.589514623	0.469476062
isovalerylcarnitine (C5)	**1.32**	**1.44**	**1.85**	0.022380322	0.120570985	0.04130779	0.204306918	0.011350164	0.135399681
beta-hydroxyisovalerate	1.08	**0.87**	**1.6**	0.418108712	0.521363947	0.085677252	0.249170401	0.008526846	0.134690715
3-methylglutaconate	**0.88**	0.96	**1.22**	0.005341269	0.063011514	0.556677081	0.473047144	0.027307626	0.157654649
N-acetylisoleucine	**0.57**	0.62	**3.21**	0.017054965	0.108145927	0.163340615	0.319562564	0.083534145	0.198054444
1-carboxyethylisoleucine	0.9	**0.74**	0.96	0.208255065	0.369101724	0.067513176	0.235121437	0.332908188	0.350141495
2-hydroxy-3-methylvalerate	**0.93**	0.9	1.04	0.038537114	0.14924289	0.439341798	0.427274271	0.934059393	0.597446399
2-methylbutyrylcarnitine (C5)	**0.8**	1.04	**1.4**	0.011993686	0.09565178	0.570860271	0.477937813	0.068532748	0.188745601
tiglylcarnitine (C5:1-DC)	**0.8**	**0.8**	0.88	0.001548861	0.039779476	0.077505627	0.244921099	0.379284134	0.376100334
3-hydroxy-2-ethylpropionate	**0.9**	0.99	**1.43**	0.063862007	0.192184883	0.885365669	0.558608522	0.065964564	0.188745601
ethylmalonate	**0.91**	**0.82**	1.07	0.06057313	0.186912842	0.035839841	0.204306918	0.316339154	0.338377979
methylsuccinate	0.95	**0.83**	**1.1**	0.425786126	0.522029768	0.015963196	0.153815112	0.097389387	0.20282035
N-acetylvaline	**0.9**	0.85	1.1	0.079090488	0.211286252	0.129617665	0.283120417	0.241025771	0.291940619

1-carboxyethylvaline	0.91	0.83	1.06	0.330825748	0.458888004	0.13157288	0.283120417	0.025555263	0.157654649
alpha-hydroxyisovalerate	0.85	0.91	1.09	0.000335036	0.023664592	0.099366123	0.259603011	0.685797423	0.516138658
isobutyrylcarnitine (C4)	0.82	0.95	1.17	0.003874929	0.058841112	0.737484904	0.524413525	0.351920898	0.36092854
methionine	1.12	1.07	1.26	0.012469937	0.09617228	0.160061167	0.318008092	0.001073161	0.067440674
N-acetylmethionine	0.87	0.83	1.16	0.010855207	0.095583799	0.015734523	0.153815112	0.025728736	0.157654649
N-formylmethionine	0.89	0.91	1.17	0.002213627	0.04443422	0.043538597	0.204306918	0.011253124	0.135399681
S-methylmethionine	1.34	1.44	1.25	0.244881209	0.401433424	0.024120673	0.185955146	0.309711829	0.333333728
methionine sulfone	1.12	1.09	1.22	0.022522129	0.120570985	0.191284905	0.337308644	0.015718841	0.147700418
methionine sulfoxide	1.17	1.06	1.24	0.001654511	0.039779476	0.420701765	0.425811742	0.003947147	0.110350299
N-acetylmethionine sulfoxide	0.83	0.78	1.16	0.034480923	0.13862355	0.339544384	0.396742525	0.854548992	0.579804408
S-adenosylhomocysteine (SAH)	1.47	1.07	1.41	0.080709609	0.214090873	0.457252902	0.431614187	0.024583598	0.157654649
cystathionine	0.93	0.94	1.4	0.138073003	0.287627054	0.497730691	0.443721097	0.011025386	0.135399681
lanthionine	0.91	0.9	1.26	0.076399293	0.20746731	0.086004523	0.24917401	0.060531745	0.187851722
cysteine sulfinic acid	0.87	0.9	0.92	0.04994494	0.169528728	0.121815941	0.280279855	0.404963606	0.385783066
hypotaurine	0.91	0.93	1.12	0.074964682	0.20746731	0.528936569	0.465045909	0.108943483	0.206018928
N-acetyltaurine	0.91	0.91	1.05	0.036350782	0.143668527	0.123325238	0.280279855	0.176683019	0.244267837
succinoyltaurine	0.86	0.85	1.28	0.093621611	0.235243938	0.177563981	0.331962142	0.022079993	0.157654649
argininosuccinate	0.76	0.79	1.05	0.041133301	0.154855191	0.122130624	0.280279855	0.847817705	0.578338356
urea	0.99	0.93	1.11	0.836627303	0.667469362	0.042405413	0.204306918	0.106891929	0.206018928
citrulline	0.96	0.97	1.17	0.482449096	0.537430697	0.655354376	0.503658993	0.095290324	0.20282035
homocitrulline	0.89	0.94	1.24	0.097910409	0.239396389	0.366630201	0.404181603	0.07345386	0.188745601
proline	1.05	0.99	1.08	0.096814785	0.238308523	0.755119111	0.527866231	0.373201257	0.372271307
dimethylarginine (SDMA + ADMA)	0.95	0.95	1.08	0.014449911	0.100309412	0.363947429	0.404181603	0.08047556	0.195452245
N-acetylarginine	0.81	0.92	1.16	0.055086761	0.177580363	0.530893412	0.465045909	0.239892631	0.291315484
N-delta-acetylornithine	0.95	0.89	0.92	0.311974296	0.448926427	0.015262135	0.153815112	0.939502986	0.600164682
trans-4-hydroxyproline	0.65	0.68	0.63	2.54638E-05	0.003673363	0.003230244	0.07775258	0.002007448	0.100923243
pro-hydroxy-pro	1.09	1.04	1.22	0.574051683	0.580117034	0.257161103	0.358698521	0.141740841	0.22479298

N-methylproline	0.72	0.93	0.97	0.002577356	0.047614135	0.717208225	0.516984541	0.815758148	0.568029937
N,N,N-trimethyl-alanylproline betaine (TMAP)	0.87	1.06	1.42	0.018993985	0.110708667	0.541409876	0.467590689	0.033353313	0.162467472
N-monomethylarginine	0.93	0.96	1.04	0.075387942	0.20746731	0.448956071	0.428925104	0.430886663	0.400417259
argininate	0.89	1.49	2.83	0.454545979	0.529761636	0.063725134	0.232405414	0.071051886	0.188745601
guanidinoacetate	1.2	1.13	1.42	0.022079095	0.120570985	0.218538303	0.348915076	0.06183715	0.188745601
creatine	1.01	0.94	1.45	0.969734363	0.695981136	0.304830304	0.3810316	0.004170423	0.110350299
creatinine	0.95	0.98	1.05	0.007464181	0.075562799	0.591726032	0.484454247	0.09843609	0.20282035
putrescine	1.22	1.06	1.29	0.188242268	0.347035211	0.435626288	0.426548407	0.097526437	0.20282035
N-acetylputrescine	1.09	0.76	1.42	0.137635913	0.287627054	0.555369399	0.473047144	0.036083577	0.162467472
N-carbamoylputrescine	1.35	1.56	1.76	0.067697806	0.198231369	0.024335447	0.185955146	0.056214865	0.187851722
N-acetyl-isoputreanine	0.91	0.92	1.11	0.004149482	0.060969448	0.407774043	0.420351028	0.108178092	0.206018928
N('1)-acetylspermidine	1.13	1.08	1.25	0.105654593	0.25183499	0.349604253	0.402681864	0.060127673	0.187851722
5-methylthioadenosine (MTA)	1.05	0.93	1.34	0.478177598	0.536817714	0.220007778	0.348915076	0.093180839	0.20282035
4-acetamidobutanoate	0.84	0.84	1.93	0.059053409	0.185194414	0.60984505	0.491926624	0.041728642	0.176292683
glutathione, oxidized (GSSG)	0.77	0.99	1.37	0.076941867	0.20746731	0.912126017	0.56658172	0.047155172	0.182661208
cysteine-glutathione disulfide	0.96	0.99	1.19	0.570702599	0.578759744	0.816624131	0.54297128	0.011861967	0.135414007
S-methylglutathione	0.95	1.04	1.8	0.486959546	0.539236729	0.686785453	0.511630265	0.02499473	0.157654649
cysteinylglycine disulfide	1.15	1.19	1.21	0.089595544	0.228759272	0.032977136	0.204306918	0.069389423	0.188745601
5-oxoproline	0.9	0.88	0.96	0.05946066	0.185463618	0.031965397	0.204306918	0.591294051	0.469620235
2-aminobutyrate	0.91	1.1	1.08	0.059826468	0.185601359	0.235584037	0.352815194	0.474348729	0.421176529
2-hydroxybutyrate/2-hydroxyisobutyrate	0.8	0.84	0.86	0.012569597	0.09617228	0.070819167	0.235121437	0.520312339	0.439532946
Ophthalmate	0.71	0.94	1.55	0.028512311	0.131620382	0.745521459	0.525680595	0.182067806	0.248242476
gamma-glutamylfelinylglycine	0.86	0.85	0.9	0.01649806	0.105776962	0.250995595	0.355735911	0.082497561	0.198054444
gamma-glutamylalanine	1	1.13	0.74	0.560843007	0.575858067	0.065733923	0.235121437	0.051904036	0.185067015
gamma-glutamylglutamate	0.96	0.87	1.4	0.570509264	0.578759744	0.140078399	0.295764293	0.066979144	0.188745601
gamma-glutamylglutamine	0.94	0.95	1.12	0.107988882	0.252450363	0.269486436	0.361110456	0.003916991	0.110350299
gamma-glutamylglycine	0.87	0.94	1.07	0.028228349	0.131360421	0.606955581	0.491926624	0.496109395	0.430770436

gamma-glutamylhistidine	0.91	1.02	1.13	0.017695583	0.109795027	0.61724764	0.49294256	0.241568806	0.291940619
gamma-glutamylisoleucine	0.85	1.02	1.05	0.039780538	0.152326081	0.908654957	0.565154847	0.64738868	0.494636556
gamma-glutamylleucine	0.88	0.98	1.1	0.04132813	0.154855191	0.673021079	0.505828125	0.324893229	0.343870965
gamma-glutamyl-epsilon-lysine	0.76	0.9	1.19	0.001980051	0.04443422	0.216544716	0.348915076	0.027532793	0.157654649
gamma-glutamylmethionine	1.08	1.15	1.47	0.479266466	0.536995375	0.076805292	0.244921099	0.009140566	0.134690715
gamma-glutamylphenylalanine	0.92	0.95	1.13	0.057259919	0.182546239	0.478171444	0.435202513	0.032437553	0.162467472
gamma-glutamylvaline	0.87	1	1.09	0.070522187	0.201453648	0.743525031	0.525680595	0.38291307	0.377465285
gamma-glutamylserine	0.98	1	1.29	0.355118449	0.480180799	0.809432914	0.542729304	0.095957644	0.20282035
gamma-glutamylcitrulline	0.86	0.88	1.22	0.153008457	0.304451536	0.3596454	0.404181603	0.103052097	0.206018928
gamma-glutamyl-2-aminobutyrate	0.88	1.26	1.23	0.064528216	0.192927031	0.09768126	0.257857023	0.126313041	0.219142586
Leucylhydroxyproline	1.68	1.27	1.52	0.002233134	0.04443422	0.012346677	0.153815112	0.072361165	0.188745601
felinylglycine	0.87	0.97	0.83	0.03194571	0.138138448	0.899807096	0.561829389	0.023318156	0.157654649
isoleucylglycine	1.33	1.12	1.93	0.049846045	0.169528728	0.469213358	0.433552866	0.073800736	0.188745601
phenylacetylalanine	2.08	1.06	1.52	0.02613998	0.127361579	0.477730689	0.435202513	0.169018975	0.243589669
phenylacetylglutamate	1.69	1.18	2.17	0.033260505	0.13862355	0.150315814	0.307925906	0.00717746	0.127932774
phenylacetylglutamine	1.83	1.18	1.99	0.1083922238	0.252450363	0.416453106	0.425811742	0.058849876	0.187851722
4-hydroxyphenylacetylglycine	0.66	0.55	0.59	0.046407894	0.164489242	0.264205739	0.358698521	0.233100674	0.287936103
phenylacetylserine	2.02	1.26	2.12	0.051869663	0.172014441	0.259573811	0.358698521	0.036338759	0.162467472
1,5-anhydroglucitol (1,5-AG)	0.65	0.86	0.73	0.019374568	0.110772471	0.633153562	0.497644204	0.148074806	0.229674679
ribonate	0.84	0.82	1.06	0.033068478	0.13862355	0.010668092	0.142657194	0.866485912	0.582620204
arabonate/xylonate	0.88	0.8	1.12	0.053793521	0.175370861	0.259760346	0.358698521	0.034855314	0.162467472
sedoheptulose	0.84	0.85	1	0.034329637	0.13862355	0.211604818	0.348915076	0.966905508	0.603880894
glucuronate	1.01	0.92	1.4	0.438954521	0.527996766	0.220663996	0.348915076	0.034853609	0.162467472
N-acetylglucosaminylasparagine	0.85	0.89	1.51	0.01183971	0.09565178	0.095240611	0.257857023	0.128809588	0.220266201
N-acetylglucosamine/N-acetylgalactosamine	0.89	0.89	1.17	0.014787458	0.100386505	0.036401097	0.204306918	0.182202973	0.248242476
citrate	0.93	0.93	1.09	0.071393467	0.202247188	0.249719931	0.355735911	0.04461504	0.18235731
aconitate [cis or trans]	0.88	0.88	1.08	0.002868733	0.047614135	0.049833642	0.213138329	0.175777522	0.244267837

isocitrate	**0.82**	0.91	**1.21**	0.000599429	0.034589023	0.127445676	0.283120417	0.077731126	0.190803079
succinylcarnitine (C4-DC)	**0.88**	0.92	1.03	0.031279568	0.136737473	0.418158037	0.425811742	0.372699166	0.372271307
fumarate	0.92	**0.82**	0.95	0.199692456	0.362943791	0.077360186	0.244921099	0.952889486	0.601051521
malate	**0.87**	**0.84**	0.98	0.005144715	0.063011514	0.069251866	0.235121437	0.881065396	0.584941523
2-methylcitrate/homocitrate	1.11	0.9	**1.34**	0.403059327	0.513831414	0.186371433	0.332913203	0.015050816	0.147700418
Malonylcarnitine	**0.58**	0.9	0.89	0.002103211	0.04443422	0.80434734	0.542319019	0.510268853	0.43480452
Malonate	**0.85**	1.51	1.2	0.013823204	0.097577835	0.818077519	0.54297128	0.207963788	0.270161686
caproate (6:0)	**0.85**	0.93	0.89	0.0214626	0.120570985	0.186717647	0.332913203	0.323558484	0.343870965
heptanoate (7:0)	**0.75**	**0.84**	0.91	0.00305163	0.047614135	0.097519575	0.257857023	0.576749851	0.463191051
caprylate (8:0)	**0.8**	0.91	0.9	0.043811921	0.158820276	0.359986565	0.404181603	0.562198149	0.458317397
pelargonate (9:0)	**0.84**	0.89	0.87	0.071500969	0.202247188	0.115073923	0.275071601	0.592920989	0.469977996
caprate (10:0)	**0.85**	**0.89**	1.01	0.01548921	0.101565763	0.058517691	0.223882146	0.881931095	0.584941523
undecanoate (11:0)	**0.87**	0.89	0.94	0.042785897	0.156969701	0.131653655	0.283120417	0.673949087	0.509770766
tetradecadienoate (14:2)	**0.74**	**0.92**	0.94	0.013866418	0.097577835	0.090533386	0.251306182	0.912296594	0.593707969
heptadecatrienoate (17:3)	1.01	0.81	0.92	0.900482562	0.681541341	0.177265694	0.331962142	0.919948115	0.593707969
stearidonate (18:4n3)	0.95	**0.84**	0.99	0.49682863	0.542966986	0.051833586	0.216981564	0.721543681	0.533821315
(11 or 12)-methyltridecanoate (a14:0 or i14:0)	**0.9**	**0.8**	0.91	0.013486856	0.097577835	0.000319368	0.021963535	0.54313695	0.44955473
branched-chain fatty acid 16:0	1.06	**0.82**	1.05	0.464856699	0.529761636	0.079845305	0.246396311	0.879911354	0.584941523
glutarate (C5-DC)	**0.81**	0.92	0.78	0.01049766	0.094648395	0.186386094	0.332913203	0.400072447	0.384577574
adipate (C6-DC)	0.87	**0.89**	0.82	0.101022384	0.243904854	0.086051743	0.24917401	0.380110164	0.376177462
Maleate	0.91	**0.77**	1.09	0.200322157	0.362943791	0.063218526	0.2323178	0.369076668	0.369623756
pimelate (C7-DC)	0.91	**0.87**	0.75	0.253973835	0.407229788	0.057273328	0.223882146	0.277679088	0.315048798
suberate (C8-DC)	0.89	**0.87**	0.83	0.165975448	0.32246912	0.045414074	0.204322457	0.408232701	0.385783066
azelate (C9-DC)	0.88	**0.9**	0.84	0.116180565	0.261298366	0.091650373	0.252119017	0.456021553	0.41530901
sebacate (C10-DC)	**0.84**	**0.86**	0.82	0.051638038	0.172014441	0.034169563	0.204306918	0.482667139	0.425715907
undecanedioate (C11-DC)	**0.84**	**0.87**	0.83	0.074570349	0.20746731	0.066145 77	0.235121437	0.47445693	0.421176529
tridecanedioate (C13-DC)	**0.84**	**0.85**	0.86	0.063372712	0.192184883	0.057660 33	0.223882146	0.554832724	0.454670441

dodecanedioate (C12-DC)	**0.83**	**0.86**	0.83	0.042980571	0.156969701	0.067854038	0.235121437	0.486685325	0.426156545
dodecadienoate (12:2)	**0.76**	0.96	1	0.010423048	0.094648395	0.378231099	0.408233249	0.873527568	0.58476811
tridecenedioate (C13:1-DC)	**0.77**	0.88	1.01	0.025952686	0.127361579	0.171109321	0.327351976	0.450437656	0.411736153
tetradecanedioate (C14-DC)	**0.86**	**0.85**	1.02	0.006303563	0.068629518	0.038699438	0.204306918	0.956854209	0.601051521
branched chain 14:0 dicarboxylic acid	0.95	0.95	**1.65**	0.448295485	0.529761636	0.574561913	0.478294057	0.030234582	0.162467472
hexadecanedioate (C16-DC)	**0.85**	**0.75**	1.22	0.010399806	0.094648395	0.000194518	0.015606967	0.100743488	0.205886978
hexadecenedioate (C16:1-DC)	0.96	**0.83**	**1.38**	0.816500948	0.665061193	0.016524144	0.153815112	0.001036353	0.067440674
heptadecanedioate (C17-DC)	**0.82**	**0.74**	1.24	0.037306982	0.146444652	0.000959551	0.057741402	0.280478161	0.315756615
octadecanedioate (C18-DC)	**0.84**	**0.83**	1.27	0.012049186	0.09565178	0.033352131	0.204306918	0.225482511	0.282620342
octadecenedioate (C18:1-DC)	0.85	**0.73**	1.24	0.143886805	0.294652872	0.008490111	0.122479891	0.304087308	0.331154014
octadecadienedioate (C18:2-DC)	**0.89**	**0.77**	**1.19**	0.054601434	0.177004693	0.006475259	0.107490126	0.035334427	0.162467472
nonadecanedioate (C19-DC)	**0.79**	0.88	1.32	0.001648726	0.039779476	0.245874836	0.355507058	0.233695307	0.287963094
eicosanedioate (C20-DC)	**0.84**	0.89	1.36	0.004636249	0.061660247	0.26118482	0.358698521	0.153660746	0.233247518
docosadioate (C22-DC)	**0.83**	**0.75**	1.35	0.000369097	0.023664592	0.016284086	0.153815112	0.304316147	0.331154014
2-aminoheptanoate	**0.88**	**0.82**	1.12	0.075230555	0.20746731	0.060080082	0.227738371	0.214368958	0.274230875
2-aminooctanoate	0.9	**0.81**	1.09	0.272116412	0.419840051	0.007876433	0.118491992	0.35811072	0.362979959
butyrylcarnitine (C4)	0.96	1.05	**1.8**	0.77743353	0.656815353	0.549599806	0.471623059	0.07069725	0.188745601
Butyrylglycine	**0.66**	**0.66**	**0.79**	0.003053071	0.047614135	0.003956983	0.086586656	0.077267719	0.190803079
propionylcarnitine (C3)	0.86	0.95	1.19	0.248917242	0.404625746	0.624201484	0.49294256	0.411789218	0.387686534
Propionylglycine	**0.63**	**0.65**	0.9	0.049931454	0.169528728	0.036554827	0.204306918	0.675308402	0.509770766
methylmalonate (MMA)	**0.9**	0.9	1.07	0.074386453	0.20746731	0.228269526	0.352181402	0.141111774	0.224503528
2-methylmalonylcarnitine (C4-DC)	0.84	**0.74**	0.91	0.310406278	0.447786748	0.016614716	0.153815112	0.639891433	0.490398867
Hexanoylglycine	**0.53**	**0.67**	0.66	0.001112598	0.036734416	0.097893193	0.257857023	0.135678374	0.223455259
3,4-methylene heptanoylglycine	**0.76**	0.7	**0.55**	0.009169267	0.08967747	0.267364611	0.359526008	0.059203155	0.187851722
N-octanoylglycine	**0.54**	0.65	0.92	0.012100831	0.09565178	0.151619971	0.30797643	0.391002219	0.379486617
N-linoleoylglycine	1.25	0.96	**0.69**	0.315832963	0.449990312	0.964305447	0.579736878	0.06036347	0.187851722
acetylcarnitine (C2)	**0.86**	1.08	1.38	0.067860033	0.198231369	0.145066552	0.300040232	0.131824161	0.222395377

octanoylcarnitine (C8)	0.9	**0.91**	1.3	0.226573184	0.389107823	0.088482736	0.251306182	0.259917579	0.305399567
decanoylcarnitine (C10)	0.93	0.93	**1.69**	0.365556495	0.488997465	0.313704168	0.382091342	0.096454951	0.20282035
laurylcarnitine (C12)	0.94	1	**1.72**	0.236329423	0.395587774	0.964615034	0.579736878	0.096176002	0.20282035
myristoylcarnitine (C14)	**0.86**	0.89	1.47	0.068020759	0.198231369	0.169656692	0.326693432	0.132550183	0.222872326
pentadecanoylcarnitine (C15)	0.92	0.87	**1.49**	0.416038453	0.521363947	0.10539397	0.265076514	0.07487908	0.188745601
palmitoylcarnitine (C16)	**0.89**	0.96	**1.39**	0.089553321	0.228759272	0.484559115	0.435202513	0.098343827	0.20282035
arachidoylcarnitine (C20)	**0.91**	0.88	1.17	0.041700804	0.155243512	0.116853811	0.277112592	0.268325717	0.311926388
behenoylcarnitine (C22)	**0.76**	0.88	1.19	0.031015681	0.136618894	0.365588115	0.404181603	0.166264006	0.243589669
lignoceroylcarnitine (C24)	**0.81**	**0.82**	**0.89**	0.002154803	0.04443422	0.019349568	0.166338451	0.054092452	0.18647039
cerotoylcarnitine (C26)	**0.62**	0.8	0.91	0.001356512	0.037273914	0.103852941	0.265076514	0.567514599	0.459719939
butenoylcarnitine (C4:1)	0.94	0.96	0.87	0.454852971	0.529761636	0.815102361	0.542729304	0.508773852	0.43480452
undecenoylcarnitine (C11:1)	1.01	**0.88**	**1.36**	0.578567513	0.581624756	0.011668402	0.151816533	0.027590065	0.157654649
palmitoleoylcarnitine (C16:1)	0.94	0.94	**1.42**	0.236950322	0.395587774	0.203430135	0.343621116	0.09288886	0.20282035
oleoylcarnitine (C18:1)	**0.85**	0.94	1.36	0.026154182	0.127361579	0.281662475	0.368909448	0.137341586	0.223455259
eicosenoylcarnitine (C20:1)	**0.88**	0.94	1.32	0.019388869	0.110772471	0.265259024	0.358698521	0.177540771	0.244541312
erucoylcarnitine (C22:1)	0.88	**0.82**	1.13	0.122712614	0.267204683	0.036954642	0.204306918	0.467163895	0.421176529
nervonoylcarnitine (C24:1)	**0.75**	**0.82**	1.29	0.004226409	0.060969448	0.018202545	0.16227356	0.176856354	0.244267837
ximenoylcarnitine (C26:1)	**0.61**	**0.72**	0.76	0.000141883	0.011695858	0.002042223	0.066390469	0.107432421	0.206018928
linoleoylcarnitine (C18:2)	**0.83**	0.91	**1.42**	0.024716161	0.127361579	0.235364696	0.352815194	0.09185847	0.20282035
linolenoylcarnitine (C18:3)	**0.81**	0.85	1.67	0.03445051	0.13862355	0.118884939	0.278350496	0.252928723	0.302893355
dihomo-linoleoylcarnitine (C20:2)	**0.83**	0.9	**1.36**	0.02566858	0.127361579	0.151247784	0.30797643	0.095197848	0.20282035
arachidonoylcarnitine (C20:4)	0.87	0.9	**1.57**	0.136376196	0.286671813	0.172856335	0.327351976	0.071488055	0.188745601
dihomo-linolenoylcarnitine (C20:3n3 or 6)	**0.77**	0.9	**1.52**	0.026673313	0.127361579	0.299420677	0.377335653	0.027480662	0.157654649
adrenoylcarnitine (C22:4)	**0.72**	**0.78**	1.3	0.013381633	0.097577835	0.019099664	0.166338451	0.280745613	0.315756615
docosapentaenoylcarnitine (C22:5n3)	0.94	0.96	**1.58**	0.457116806	0.529761636	0.434867034	0.426548407	0.010443828	0.135399681
docosahexaenoylcarnitine (C22:6)	0.87	0.93	**1.44**	0.118458478	0.263916849	0.214828997	0.348915076	0.000849103	0.067440674
adipoylcarnitine (C6-DC)	**0.81**	**0.8**	1.05	0.004669152	0.061660247	0.04028802	0.204306918	0.541073564	0.448880452

suberoylcarnitine (C8-DC)	**0.69**	0.68	**0.52**	0.004866394	0.062401572	0.189932307	0.336154831	0.093796602	0.20282035
octadecenedioylcarnitine (C18:1-DC)	0.87	0.87	**1.37**	0.456415095	0.529761636	0.186090388	0.332913203	0.087649995	0.201212418
(R)-3-hydroxybutyrylcarnitine	**0.38**	1.03	1.68	0.012666679	0.09617228	0.395444189	0.420239057	0.128742785	0.220266201
(S)-3-hydroxybutyrylcarnitine	**0.75**	0.98	1.15	0.08813053	0.227435258	0.49290072	0.440230476	0.826614824	0.571631011
3-hydroxyoleoylcarnitine	**0.81**	**0.89**	1.23	0.002878274	0.047614135	0.070446038	0.235121437	0.435150625	0.402149668
Deoxycarnitine	0.99	1.09	**1.47**	0.764546462	0.654573022	0.450632964	0.428925104	0.059612122	0.187851722
Carnitine	1.04	**1.13**	**1.39**	0.571789709	0.5788449	0.075777383	0.244921099	0.047501402	0.182661208
3-hydroxybutyrate (BHBA)	**0.78**	1	1.1	0.026117999	0.127361579	0.930534095	0.569214603	0.660238277	0.502164771
palmitoylcholine	0.88	1.12	**1.3**	0.537148913	0.564331903	0.837809041	0.549409701	0.05977979	0.187851722
Oleoylcholine	0.92	1.14	**1.33**	0.624379038	0.6048875	0.848816351	0.549409701	0.077156659	0.190803079
Stearoylcholine	0.95	1.18	**1.35**	0.707907608	0.63254422	0.717372298	0.516984541	0.050755446	0.185067015
docosahexaenoylcholine	0.9	1.29	**1.52**	0.48532498	0.539236729	0.375596981	0.406659615	0.025116347	0.157654649
Arachidonoylcholine	0.98	1.21	**1.44**	0.787332634	0.660460167	0.575259939	0.478294057	0.035422141	0.162467472
alpha-hydroxycaproate	0.89	**0.75**	1.04	0.424228352	0.522029768	0.083187747	0.247203017	0.708095756	0.527578826
2-hydroxyheptanoate	0.87	0.9	**0.83**	0.136620841	0.286671813	0.471844799	0.435149097	0.037858214	0.165857635
2-hydroxyoctanoate	**0.72**	**0.87**	1.13	0.008941215	0.088954776	0.001548229	0.066390469	0.289245492	0.321717856
2-hydroxydecanoate	**0.85**	**0.77**	1.17	0.047131318	0.165110446	0.028578541	0.201009166	0.308776561	0.333333728
2-hydroxylaurate	**0.83**	**0.85**	1.07	0.006892233	0.071018685	0.006231623	0.107140226	0.839052524	0.576407059
2-hydroxymyristate	1.1	**0.77**	0.85	0.682364248	0.625988494	0.036930672	0.204306918	0.472512964	0.421176529
3-hydroxyhexanoate	**0.82**	1.04	1	0.040125192	0.152326081	0.956513808	0.578975712	0.945852339	0.601051521
3-hydroxyoctanoate	**0.75**	1.11	0.86	0.011005389	0.095583799	0.763877654	0.529875394	0.733659348	0.540033566
3-hydroxydecanoate	**0.78**	1	0.99	0.002783765	0.047614135	0.608986276	0.491926624	0.809569265	0.565585765
3-hydroxysebacate	**0.76**	0.89	1	0.012898615	0.096661401	0.222422835	0.348915076	0.911154224	0.593707969
3-hydroxylaurate	**0.79**	0.96	0.94	0.032078839	0.138138448	0.273043618	0.361110456	0.77601509	0.552868188
3-hydroxymyristate	**0.85**	0.92	1.09	0.09560676	0.236773672	0.171542969	0.327351976	0.521884546	0.439532946
8-hydroxyoctanoate	0.88	**0.85**	0.81	0.156325922	0.307867688	0.035305426	0.204306918	0.353370236	0.36092854
16-hydroxypalmitate	**0.85**	**0.85**	1.12	0.001347602	0.037273914	0.087544751	0.250859325	0.407860772	0.385783066

13-HODE + 9-HODE	0.82	0.77	1.09	0.122451405	0.267204683	0.00916044	0.125996273	0.609904233	0.479102764
hydroxy-undecanedioate (OH-C11:0-DC)	0.85	0.93	0.86	0.095118291	0.236579324	0.313934358	0.382091342	0.516074281	0.437751815
9,10-DiHOME	0.92	0.82	1.11	0.679029522	0.625988494	0.062759596	0.2323178	0.397932594	0.383253396
2S,3R-dihydroxybutyrate	0.9	1	0.97	0.093431313	0.235243938	0.973726105	0.58081652	0.995067307	0.613068922
Azelaoyltaurine	0.81	0.77	1.24	0.032787174	0.13862355	0.030337195	0.204306918	0.083910671	0.198054444
Hexanoyltaurine	0.75	0.83	1.14	0.005350757	0.063011514	0.069938509	0.235121437	0.166690376	0.243589669
myo-inositol	1.38	0.97	1.35	0.018262408	0.11002535	0.991632218	0.587177628	0.468939737	0.421176529
choline phosphate	0.98	1.01	1.19	0.679626961	0.625988494	0.725235634	0.519117628	0.073796119	0.188745601
glycerophosphorylcholine (GPC)	0.97	0.95	1.16	0.742189107	0.644011559	0.590955643	0.484454247	0.003667854	0.110350299
Glycerophosphoethanolamine	0.92	0.9	1.06	0.040050553	0.152326081	0.119110433	0.278350496	0.016745942	0.147700418
1-myristoyl-2-palmitoyl-GPC (14:0/16:0)	0.96	0.96	1.21	0.839426516	0.667469362	0.300888769	0.37819573	0.081629999	0.197302894
1-myristoyl-2-arachidonoyl-GPC (14:0/20:4)	1.01	0.88	1.68	0.627674996	0.6048875	0.079295761	0.246279175	0.042805635	0.177853564
1,2-dipalmitoyl-GPC (16:0/16:0)	1.01	0.94	1.11	0.562710461	0.575858067	0.145219854	0.300040232	0.016672231	0.147700418
1-palmitoyl-2-palmitoleoyl-GPC (16:0/16:1)	0.98	0.91	1.27	0.820442099	0.665061193	0.013775891	0.153815112	0.020197752	0.149327958
1-palmitoyl-2-stearoyl-GPC (16:0/18:0)	1.07	0.92	1.17	0.068363569	0.198231369	0.115421764	0.275071601	0.091790917	0.20282035
1-palmitoyl-2-oleoyl-GPC (16:0/18:1)	0.95	0.93	1.15	0.181750528	0.340547669	0.028810814	0.201009166	0.073094458	0.188745601
1-palmitoyl-2-linoleoyl-GPC (16:0/18:2)	0.96	0.94	1.05	0.027279975	0.127979256	0.039530621	0.204306918	0.243526799	0.292898724
1-palmitoyl-2-arachidonoyl-GPC (16:0/20:4n6)	0.98	0.98	1.2	0.814316151	0.665061193	0.601730479	0.490144289	0.049273257	0.184629535
1-palmitoleoyl-2-linoleoyl-GPC (16:1/18:2)	0.98	0.9	1.28	0.930452924	0.684824161	0.023896959	0.185955146	0.034598987	0.162467472
1-palmitoleoyl-2-linolenoyl-GPC (16:1/18:3)	1.06	0.88	1.55	0.469550514	0.533358647	0.036597042	0.204306918	0.019982069	0.149327958
1-stearoyl-2-oleoyl-GPC (18:0/18:1)	1.02	0.95	1.19	0.453111603	0.529761636	0.262188217	0.358698521	0.033400916	0.162467472
1-stearoyl-2-linoleoyl-GPC (18:0/18:2)	1.01	0.96	1.06	0.488422007	0.539236729	0.272868421	0.361110456	0.058459601	0.187851722
1-stearoyl-2-arachidonoyl-GPC (18:0/20:4)	1.05	1.01	1.15	0.049591122	0.169528728	0.864466801	0.553985264	0.034610157	0.162467472
1-stearoyl-2-docosahexaenoyl-GPC (18:0/22:6)	1.02	1.03	1.2	0.457572935	0.529761636	0.71662166	0.516984541	0.096654019	0.20282035
1-oleoyl-2-linoleoyl-GPC (18:1/18:2)	1.03	0.96	1.1	0.172961252	0.331575976	0.289724819	0.369959145	0.051216764	0.185067015
1-oleoyl-2-docosahexaenoyl-GPC (18:1/22:6)	1.13	1.11	1.33	0.018891396	0.110708667	0.308125707	0.382091342	0.028721895	0.158522177
1,2-dilinoleoyl-GPC (18:2/18:2)	1.01	0.91	1.21	0.528620833	0.559463841	0.090391307	0.251306182	0.040253177	0.172966222

1-linoleoyl-2-linolenoyl-GPC (18:2/18:3)	1.04	0.89	1.44	0.491002244	0.540695708	0.025410905	0.18943245	0.036517233	0.162467472
1-linoleoyl-2-arachidonoyl-GPC (18:2/20:4n6)	1.12	0.98	1.4	0.101831383	0.244833664	0.634980393	0.497853267	0.035263769	0.162467472
1,2-dilinolenoyl-GPC (18:3/18:3)	1.07	0.77	2.62	0.461856501	0.529761636	0.013142326	0.153815112	0.10590363	0.206018928
1-palmitoyl-2-arachidonoyl-GPE (16:0/20:4)	0.86	0.71	1.08	0.213800305	0.371595946	0.072063864	0.235998708	0.522812083	0.439532946
1-palmitoyl-2-docosahexaenoyl-GPE (16:0/22:6)	0.92	0.77	1.34	0.318047489	0.451591034	0.339328603	0.396742525	0.069677531	0.188745601
1-stearoyl-2-oleoyl-GPE (18:0/18:1)	1.18	0.82	1.7	0.164240637	0.32017664	0.045016293	0.204322457	0.016622983	0.147700418
1-stearoyl-2-linoleoyl-GPE (18:0/18:2)	0.97	0.77	1.3	0.710162714	0.63254422	0.061806099	0.230648714	0.034212298	0.162467472
1-stearoyl-2-arachidonoyl-GPE (18:0/20:4)	0.96	0.69	1.21	0.421014169	0.522029768	0.005741126	0.102362942	0.063120977	0.188745601
1-stearoyl-2-docosahexaenoyl-GPE (18:0/22:6)	1.01	0.78	1.23	0.649088605	0.618062071	0.030839836	0.204306918	0.012120739	0.135414007
1-palmitoyl-2-linoleoyl-GPI (16:0/18:2)	0.97	0.87	0.71	0.383331326	0.49914771	0.016546384	0.153815112	0.106579258	0.206018928
1-palmitoyl-2-arachidonoyl-GPI (16:0/20:4)	0.82	0.85	1.21	0.021590722	0.120570985	0.186562208	0.332913203	0.069002179	0.188745601
1-stearoyl-2-arachidonoyl-GPI (18:0/20:4)	0.97	0.94	1.22	0.653442558	0.620160317	0.42130405	0.425811742	0.046034216	0.182661208
1-palmitoyl-GPA (16:0)	0.67	0.87	1.35	0.061867292	0.189890828	0.56575398	0.477099929	0.204577795	0.267561383
1-linoleoyl-GPA (18:2)	0.67	0.78	0.87	4.6544E-05	0.004476239	0.113621475	0.274863304	0.075086149	0.188745601
1-palmitoleoyl-GPC (16:1)	0.97	0.88	1.4	0.90365968	0.681541341	0.103781257	0.265076514	0.051514701	0.185067015
1-stearoyl-GPC (18:0)	1.02	0.97	1.23	0.560709312	0.575858067	0.534943966	0.465685354	0.064633175	0.188745601
1-oleoyl-GPC (18:1)	1.03	0.94	1.39	0.452488019	0.529761636	0.313040751	0.382091342	0.023068279	0.157654649
1-linoleoyl-GPC (18:2)	1.02	0.96	1.38	0.671616344	0.625988494	0.368437121	0.404181603	0.006652064	0.127932774
1-linolenoyl-GPC (18:3)	1.05	0.88	1.49	0.538674567	0.564331903	0.090762625	0.251306182	0.030888253	0.162467472
1-arachidonoyl-GPC (20:4n6)	1.09	1.07	1.65	0.286074065	0.429880746	0.516009475	0.457474893	0.008899896	0.134690715
1-palmitoyl-GPE (16:0)	0.9	0.84	1.26	0.116377517	0.261298366	0.001688895	0.066390469	0.110213375	0.206018928
1-stearoyl-GPE (18:0)	0.91	0.86	1.4	0.222183121	0.383853344	0.003818577	0.086586656	0.064463754	0.188745601
1-oleoyl-GPE (18:1)	1.14	0.97	1.63	0.10918689	0.252450363	0.299063733	0.377335653	0.002889141	0.110350299
1-linoleoyl-GPE (18:2)	1.05	0.92	1.43	0.421496161	0.522029768	0.245231772	0.355507058	0.006390793	0.127932774
1-arachidonoyl-GPE (20:4n6)	0.96	0.83	1.41	0.724998222	0.637725575	0.00128323	0.066390469	0.025049743	0.157654649
1-(1-enyl-palmitoyl)-2-oleoyl-GPE (P-16:0/18:1)	1.06	0.89	1.13	0.169183154	0.327349933	0.023059923	0.185955146	0.359973894	0.364134325
1-(1-enyl-palmitoyl)-2-palmitoyl-GPC (P-16:0/16:0)	0.93	0.89	1.36	0.143901043	0.294652872	0.052802772	0.217260251	0.070573125	0.188745601

1-(1-enyl-stearoyl)-2-oleoyl-GPE (P-18:0/18:1)	0.95	0.76	1.13	0.784648869	0.660012209	0.002482383	0.066390469	0.188476357	0.254035873
1-(1-enyl-stearoyl)-2-linoleoyl-GPE (P-18:0/18:2)	0.98	0.81	1.12	0.935496571	0.686784343	0.02292068	0.185955146	0.17630944	0.244267837
1-(1-enyl-palmitoyl)-2-linoleoyl-GPC (P-16:0/18:2)	0.94	0.9	1.21	0.24741706	0.404441459	0.042965123	0.204306918	0.093524163	0.20282035
1-(1-enyl-stearoyl)-2-arachidonoyl-GPE (P-18:0/20:4)	1.01	0.96	1.32	0.795758861	0.663034033	0.479586873	0.435202513	0.087131071	0.200938688
1-(1-enyl-palmitoyl)-GPC (P-16:0)	1	0.93	1.4	0.770136511	0.655448768	0.238921626	0.352815194	0.06751232	0.188745601
1-(1-enyl-palmitoyl)-GPE (P-16:0)	0.98	0.95	1.31	0.802652969	0.663192571	0.46022315	0.431678512	0.093288096	0.20282035
1-(1-enyl-oleoyl)-GPE (P-18:1)	0.84	0.88	1.37	0.731058442	0.639316219	0.297909415	0.377335653	0.085315895	0.198827981
1-(1-enyl-stearoyl)-GPE (P-18:0)	1	0.92	1.3	0.591216762	0.587045929	0.306977992	0.381861341	0.053751411	0.18647039
glycerol 3-phosphate	0.91	0.86	1.12	0.022566584	0.120570985	0.068821716	0.235121437	0.44293486	0.407098533
palmitoleoyl-linoleoyl-glycerol (16:1/18:2)	0.96	0.57	1.68	0.900486222	0.681541341	0.00783255	0.118491992	0.11686119	0.211023415
oleoyl-linoleoyl-glycerol (18:1/18:2)	0.89	0.66	1.65	0.260178078	0.414727527	0.016177623	0.153815112	0.070241906	0.188745601
oleoyl-linoleoyl-glycerol (18:1/18:2)	0.93	0.63	1.52	0.577861619	0.581624756	0.00470861	0.094447584	0.288629514	0.321717856
linoleoyl-linoleoyl-glycerol (18:2/18:2)	0.8	0.68	1.65	0.047212583	0.165110446	0.049274186	0.213138329	0.078049137	0.190803079
oleoyl-arachidonoyl-glycerol (18:1/20:4)	0.83	0.76	1.9	0.077945681	0.209196458	0.159527122	0.318008092	0.708343851	0.527578826
oleoyl-arachidonoyl-glycerol (18:1/20:4)	0.85	0.81	1.95	0.296494396	0.439812551	0.082696801	0.247203017	0.448020439	0.410272573
linoleoyl-arachidonoyl-glycerol (18:2/20:4)	0.85	0.88	1.74	0.108848577	0.252450363	0.467056248	0.433222741	0.047058386	0.182661208
Sphinganine	1	1.1	1.16	0.978771956	0.698989881	0.040913916	0.204306918	0.253055026	0.302893355
N-palmitoyl-sphinganine (d18:0/16:0)	1.2	0.91	1.73	0.053770054	0.175370861	0.261119902	0.358698521	0.009644798	0.134690715
N-palmitoyl-sphingosine (d18:1/16:0)	0.89	0.88	1.57	0.462885233	0.529761636	0.172131322	0.327351976	0.031151135	0.162467472
N-palmitoyl-phytosphingosine (t18:0/16:0)	0.91	0.93	1.89	0.65774062	0.620160317	0.386405321	0.412454431	0.024203835	0.157654649
N-stearoyl-sphingosine (d18:1/18:0)	0.96	0.93	1.45	0.66740909	0.625988494	0.313112332	0.382091342	0.023417633	0.157654649
N-palmitoyl-heptadecasphingosine (d17:1/16:0)	0.9	0.73	2.04	0.925503461	0.682923451	0.076140082	0.244921099	0.024680413	0.157654649
ceramide (d18:1/17:0, d17:1/18:0)	0.9	0.74	2.17	0.847515392	0.667630561	0.28431475	0.368922293	0.034695739	0.162467472
ceramide (d18:1/20:0, d16:1/22:0, d20:1/18:0)	0.98	0.9	1.51	0.997529358	0.705511689	0.188784244	0.335355838	0.002504733	0.106708392
ceramide (d18:2/24:1, d18:1/24:2)	0.94	0.87	1.72	0.455399193	0.529761636	0.195604654	0.342295303	0.038518537	0.166939378
glycosyl-N-palmitoyl-sphingosine (d18:1/16:0)	0.97	0.88	1.32	0.827708615	0.665061193	0.060865888	0.228914557	0.065467106	0.188745601
glycosyl-N-stearoyl-sphingosine (d18:1/18:0)	0.95	0.88	1.37	0.627016734	0.6048875	0.050109085	0.213138329	0.126058836	0.219142586

glycosyl ceramide (d18:1/20:0, d16:1/22:0)	0.95	**0.89**	1.33	0.578285419	0.581624756	0.025577513	0.18943245	0.170773679	0.243589669
glycosyl ceramide (d18:2/24:1, d18:1/24:2)	0.98	0.93	**1.49**	0.733089515	0.639316219	0.250956326	0.355735911	0.067459305	0.188745601
myristoyl dihydrosphingomyelin (d18:0/14:0)	**1.14**	1.03	**1.54**	0.080882379	0.214090873	0.929636214	0.569214603	0.035952499	0.162467472
palmitoyl dihydrosphingomyelin (d18:0/16:0)	**1.13**	0.98	**1.27**	0.018304727	0.11002535	0.456363078	0.431614187	0.057255352	0.187851722
behenoyl dihydrosphingomyelin (d18:0/22:0)	1.03	0.94	**1.35**	0.301869456	0.443228168	0.22350618	0.349339937	0.085599838	0.198827981
sphingomyelin (d18:0/18:0, d19:0/17:0)	1.1	0.94	**1.38**	0.196306282	0.359603885	0.212072199	0.348915076	0.097947326	0.20282035
sphingomyelin (d18:0/20:0, d16:0/22:0)	1.07	**0.89**	**1.41**	0.210332585	0.370027028	0.044410057	0.204306918	0.068778277	0.188745601
sphingomyelin (d17:1/14:0, d16:1/15:0)	1	0.94	**1.51**	0.438754124	0.527996766	0.349646298	0.402681864	0.037939037	0.165857635
sphingomyelin (d18:1/14:0, d16:1/16:0)	1.01	0.95	**1.3**	0.604751393	0.594483094	0.364710491	0.404181603	0.094939192	0.20282035
sphingomyelin (d18:2/14:0, d18:1/14:1)	1.09	0.98	**1.52**	0.149188836	0.30003244	0.539713528	0.467590689	0.089194022	0.20282035
sphingomyelin (d17:1/16:0, d18:1/15:0, d16:1/17:0)	0.96	0.91	**1.33**	0.943672267	0.68971725	0.234276313	0.352815194	0.09087638	0.20282035
sphingomyelin (d17:2/16:0, d18:2/15:0)	0.98	0.9	**1.5**	0.537382431	0.564331903	0.235767234	0.352815194	0.047596018	0.182661208
sphingomyelin (d18:1/20:0, d16:1/22:0)	1	**0.91**	1.22	0.781798907	0.658574968	0.083033354	0.247203017	0.105496161	0.206018928
sphingomyelin (d18:1/20:1, d18:2/20:0)	1.01	0.97	**1.2**	0.706227629	0.63254422	0.422118686	0.425811742	0.073280346	0.188745601
sphingomyelin (d18:1/21:0, d17:1/22:0, d16:1/23:0)	0.92	0.93	**1.34**	0.473864625	0.53614819	0.315099915	0.382091342	0.078181788	0.190803079
sphingomyelin (d18:2/21:0, d16:2/23:0)	0.94	0.93	**1.4**	0.710338192	0.63254422	0.483136691	0.435202513	0.093295511	0.20282035
sphingomyelin (d18:1/22:1, d18:2/22:0, d16:1/24:1)	1.02	**0.91**	1.28	0.525412226	0.557316635	0.081722216	0.247203017	0.109423829	0.206018928
sphingomyelin (d18:1/22:2, d18:2/22:1, d16:1/24:2)	0.98	**0.92**	1.24	0.800282841	0.663192571	0.058499779	0.223882146	0.170149309	0.243589669
sphingomyelin (d18:2/23:0, d18:1/23:1, d17:1/24:1)	0.93	0.93	**1.37**	0.552447826	0.57231721	0.288264179	0.36907298	0.083273807	0.198054444
sphingomyelin (d18:1/24:1, d18:2/24:0)	1.03	**0.92**	1.3	0.495237552	0.54225515	0.09802134	0.257857023	0.10938297	0.206018928
sphingomyelin (d18:2/24:1, d18:1/24:2)	1.01	**0.91**	1.28	0.587341406	0.586087964	0.052416664	0.217260251	0.124010852	0.218026177
sphingosine 1-phosphate	1.02	1.04	**1.42**	0.717283583	0.635432938	0.775002151	0.533096551	0.01906575	0.149327958
3-hydroxy-3-methylglutarate	**0.92**	0.93	1.13	0.011835123	0.09565178	0.225047124	0.349479085	0.148725489	0.229674679
Cholesterol	1.01	0.93	**1.34**	0.905535913	0.681541341	0.251114631	0.355735911	0.015600637	0.147700418
beta-sitosterol	0.96	**0.77**	**1.37**	0.643543714	0.615800262	0.005387656	0.102362942	0.06653482	0.188745601
Campesterol	0.93	**0.86**	**1.35**	0.187224133	0.347035211	0.020161739	0.170279576	0.002547023	0.106708392
5alpha-pregnan-3alpha,20beta-diol disulfate	**0.63**	0.8	0.66	0.030221029	0.135182436	0.450480793	0.428925104	0.111906907	0.206082563

Taurocholate	0.85	**1.89**	1.32	0.472985265	0.53614819	0.086439012	0.24917401	0.327586281	0.345884915
Deoxycholate	1.45	0.9	**1.87**	0.432814259	0.527169475	0.843084398	0.549409701	0.0729596	0.188745601
taurolithocholate 3-sulfate	0.89	0.93	**1.23**	0.315489448	0.449990312	0.843603129	0.549409701	0.058844049	0.187851722
N1-methylinosine	**0.94**	0.98	1.04	0.056600128	0.181445265	0.903581476	0.562726338	0.635161329	0.487927107
Urate	0.82	0.91	**1.19**	0.238588932	0.396286516	0.401606355	0.420351028	0.011170651	0.135399681
Allantoin	**0.88**	0.89	1.02	0.076649013	0.20746731	0.232599777	0.352815194	0.485826079	0.426156545
allantoic acid	**0.64**	0.82	0.79	0.053752295	0.175370861	0.212760975	0.348915076	0.259995112	0.305399567
1-methylhypoxanthine	**0.84**	**0.77**	1.11	0.030488942	0.135331759	0.012831403	0.153815112	0.196678548	0.262914591
N1-methyladenosine	0.96	**0.9**	**1.16**	0.191814596	0.352494816	0.039035055	0.204306918	0.005393902	0.127932774
N6-carbamoylthreonyladenosine	**0.88**	0.9	1.06	0.026927593	0.127361579	0.263064438	0.358698521	0.464924343	0.421149515
N6-succinyladenosine	**0.79**	0.82	0.95	0.018733758	0.110708667	0.437709383	0.426548407	0.551954338	0.454056179
7-methylguanine	**0.94**	0.93	**1.16**	0.045976293	0.164489242	0.123667099	0.280279855	0.027432435	0.157654649
N2,N2-dimethylguanosine	0.84	0.95	**1.36**	0.106052672	0.25183499	0.415395887	0.425811742	0.066391818	0.188745601
N2,N2-dimethylguanine	1.02	0.92	**1.06**	0.563849463	0.575858067	0.407554544	0.420351028	0.054028857	0.18647039
Uracil	0.9	**0.81**	1.29	0.518097528	0.554617586	0.089896064	0.251306182	0.434349828	0.402148846
5,6-dihydrouridine	**0.94**	**0.88**	**1.08**	0.03459384	0.13862355	0.047371729	0.209219498	0.054152154	0.18647039
2'-O-methyluridine	**0.92**	0.95	1.12	0.05084695	0.171326585	0.475915174	0.435202513	0.113692878	0.208607394
2'-deoxyuridine	**0.75**	0.77	1.02	0.042269016	0.156350139	0.254376428	0.358063602	0.880953472	0.584941523
3-ureidoisobutyrate	**0.92**	0.89	**1.09**	0.014602265	0.100309412	0.118548862	0.278350496	0.055452665	0.187851722
3-ureidopropionate	**0.93**	**0.95**	1.06	0.011399815	0.09565178	0.045986634	0.204982734	0.130095511	0.220962005
N-acetyl-beta-alanine	0.96	0.98	**1.3**	0.542887735	0.567507603	0.788631905	0.535491275	0.01023243	0.135399681
3-(3-amino-3-carboxypropyl)uridine	**0.78**	**0.79**	**1.17**	0.00957281	0.091953548	0.031761052	0.204306918	0.092648042	0.20282035
Cytidine	**0.85**	0.93	1.32	0.088288691	0.227435258	0.2881486	0.36907298	0.14008465	0.224288963
5-methylcytidine	0.93	0.96	**1.11**	0.210162159	0.370027028	0.579093959	0.480651673	0.059082732	0.187851722
N4-acetylcytidine	**0.93**	0.95	0.95	0.026727862	0.127361579	0.450840128	0.428925104	0.845329946	0.578210425
2'-deoxycytidine	**0.92**	**0.93**	**1.09**	0.02536979	0.127361579	0.070740726	0.235121437	0.019693262	0.149327958
5-methyl-2'-deoxycytidine	**1.15**	1	**1.2**	0.006538434	0.069868387	0.690650206	0.512603558	0.08575806	0.198827981

5-hydroxymethylcytidine	0.94	1.34	0.99	0.04878818	0.169528728	0.328016764	0.391130235	0.85866711	0.580910098
5-hydroxymethyl-2'-deoxycytidine	0.94	0.98	1.11	0.069385861	0.199193727	0.600692408	0.490128039	0.083906457	0.198054444
3-aminoisobutyrate	1.31	1.24	1.12	0.126895725	0.272204588	0.017934198	0.16227356	0.389177436	0.379486617
nicotinate ribonucleoside	0.56	0.68	1.15	0.384070827	0.49914771	0.043763735	0.204306918	0.535968056	0.446857032
Nicotinamide	0.9	0.89	1.19	0.098836142	0.240640195	0.00209292	0.066390469	0.189101515	0.254196992
nicotinamide riboside	0.87	0.8	1.03	0.709813864	0.63254422	0.044561824	0.204306918	0.892017901	0.588577333
trigonelline (N'-methylnicotinate)	1.04	1.03	1.25	0.403383493	0.513831414	0.736086744	0.524413525	0.042470553	0.177853564
N1-Methyl-2-pyridone-5-carboxamide	1.13	0.94	1.13	0.093766033	0.235243938	0.625258641	0.49294256	0.347914857	0.36092854
Pantothenate	0.93	1.04	0.97	0.05898889	0.185194414	0.178598951	0.331962142	0.672062467	0.509616072
ascorbic acid 3-sulfate	0.82	0.81	1.16	0.087149546	0.227122484	0.434971366	0.426548407	0.220244025	0.278848593
Threonate	0.91	0.9	1.17	0.092928838	0.235243938	0.153475364	0.310435324	0.184657381	0.250230224
oxalate (ethanedioate)	0.92	0.93	1	0.085207377	0.224509031	0.235607146	0.352815194	0.946197416	0.601051521
gulonate	0.92	0.77	1.1	0.005350159	0.063011514	0.180289574	0.332536653	0.144060828	0.226280918
alpha-tocopherol	1.1	0.97	1.06	0.015982792	0.103624719	0.465500423	0.433222741	0.203121093	0.267324439
alpha-tocotrienol	1.07	0.99	1.43	0.613020585	0.599547752	0.89354578	0.560606892	0.015027184	0.147700418
gamma-tocotrienol	1.13	1.32	0.44	0.099784042	0.241927294	0.252221973	0.356072109	0.05405708	0.18647039
gamma-tocopherol/beta-tocopherol	1.14	0.99	1.39	0.181772381	0.340547669	0.621283859	0.49294256	0.008564548	0.134690715
Pterin	0.93	0.78	1.16	0.612392356	0.599547752	0.096731352	0.257857023	0.099526169	0.204229374
Heme	0.46	0.59	0.79	0.426103836	0.522029768	0.031002096	0.204306918	0.65004557	0.495912883
Biliverdin	0.65	0.95	1.07	0.07633406	0.20746731	0.667193393	0.505810021	0.866589079	0.582620204
retinol (Vitamin A)	0.96	0.97	1.26	0.124248442	0.269531822	0.654975152	0.503658993	0.074075683	0.188745601
carotene diol (1)	1.02	0.95	1.36	0.914625791	0.681996086	0.423524874	0.425811742	0.048031982	0.182937852
carotene diol (2)	1.07	0.97	1.21	0.273151636	0.420313434	0.644463444	0.50117957	0.154030992	0.233247518
beta-cryptoxanthin	0.95	0.78	1.26	0.592099008	0.587045929	0.129851653	0.283120417	0.011580814	0.135399681
pyridoxine (Vitamin B6)	0.68	0.78	1.12	0.589101412	0.586087964	0.050472753	0.213138329	0.625779261	0.482836437
Pyridoxamine	0.94	0.59	1.04	0.359060514	0.482959916	0.038241344	0.204306918	0.972515469	0.606223646
Pyridoxal	1.07	1	1.23	0.264478206	0.416974539	0.820131098	0.54297128	0.012475654	0.136349172

Pyridoxate	0.99	0.9	1.46	0.655590677	0.620160317	0.397729529	0.420351028	0.018516066	0.149327958
4-allylcatechol sulfate	3.58	2.86	2.92	3.53676E-05	0.004081657	8.14972E-06	0.000980826	0.014412119	0.147700418
4-acetylphenol sulfate	1.65	0.97	1.48	0.076750277	0.20746731	0.466934125	0.433222741	0.405888327	0.385783066
4-ethylphenylsulfate	1.31	0.99	2.57	0.236785736	0.395587774	0.796943771	0.539898054	0.027704806	0.157654649
Theophylline	35.37	44.08	42.57	8.74E-12	5.05E-09	8E-11	3.807E-08	1.23162E-05	0.006191873
2-oxindole-3-acetate	0.95	0.78	1.58	0.518909392	0.554617586	0.077840965	0.244921099	0.019074105	0.149327958
beta-guanidinopropanoate	1.18	1.23	1.71	0.097052495	0.238308523	0.122870609	0.280279855	0.018788592	0.149327958
dihydrocaffeate sulfate	1.66	0.42	2.65	0.276658986	0.422331731	0.410983689	0.422754413	0.07179109	0.188745601
Cinnamoylglycine	0.55	0.65	2.72	0.044037758	0.158820276	0.930553503	0.569214603	0.175384588	0.244267837
Ergothioneine	1.25	1.18	1.49	0.005867643	0.067716481	0.02800892	0.201009166	0.0073796	0.127932774
Erythritol	0.99	0.81	0.86	0.907534913	0.681541341	0.389685355	0.41503533	0.019939828	0.149327958
Homostachydrine	0.96	0.97	1.29	0.42427851	0.522029768	0.571869867	0.47795185	0.028222942	0.157654649
indolin-2-one	0.99	0.7	1.64	0.775840453	0.656815353	0.0152712	0.153815112	0.074782827	0.188745601
Mannonate	0.89	0.85	0.93	0.026567253	0.127361579	0.055536039	0.222793784	0.616153117	0.481005215
methyl indole-3-acetate	0.9	0.8	2.07	0.314928704	0.449990312	0.354077028	0.404181603	0.000966897	0.067440674
Stachydrine	0.92	0.85	1.13	0.038351725	0.14924289	0.127826271	0.283120417	0.209544061	0.270815026
Tartarate	0.59	0.93	1.22	0.033579036	0.13862355	0.841377193	0.549409701	0.255099229	0.303190633
4-vinylguaiacol sulfate	0.85	0.84	0.83	0.229877743	0.390138436	0.98942516	0.587177628	0.065148978	0.188745601
Pyrraline	0.9	0.76	1.04	0.279553664	0.424504525	0.071327984	0.235188731	0.844964059	0.578210425
eugenol sulfate	33.99	28.82	21.9	7.06E-09	2.036469E-06	9.6877E-07	0.000233185	0.005835333	0.127932774
(2,4 or 2,5)-dimethylphenol sulfate	0.6	0.71	0.73	0.001079852	0.036734416	0.148169362	0.304825975	0.313451737	0.336004304
tartronate (hydroxymalonate)	0.94	0.87	1.01	0.23467803	0.395587774	0.090832863	0.251306182	0.847009083	0.578338356
(S)-a-amino-omega-caprolactam	0.97	0.94	1.12	0.320497386	0.451591034	0.213807277	0.348915076	0.058780707	0.187851722
Salicylate	1.43	0.9	1.57	0.094661156	0.236461563	0.95978599	0.579003158	0.229414347	0.285486917
2,6-dihydroxybenzoic acid	1.08	0.82	1.19	0.830702428	0.665061193	0.283388183	0.368909448	0.028162975	0.157654649
sulfate	0.93	0.9	1.05	0.01505201	0.10099428	0.001725458	0.066390469	0.018952995	0.149327958
O-sulfo-L-tyrosine	0.94	0.87	1.22	0.120478986	0.26482994	0.041578312	0.204306918	0.041006301	0.174709125

Ectoine		1.15	1.09	**1.74**		0.278485031	0.42399758	0.783644856	0.535491275	0.048847657	0.184629535	
1,2,3-benzenetriol sulfate (2)		**11.31**	**2.23**	**15.29**		0.001202898	0.036734416	0.013660554	0.153815112	0.009361435	0.134690715	
3-hydroxypyridine sulfate		1.16	**0.67**	1.95		0.618504072	0.601850467	0.048285281	0.21131557	0.136400431	0.223455259	
6-hydroxyindole sulfate		0.72	0.65	**1.8**		0.769824817	0.655448768	0.716362442	0.516984541	0.059780856	0.187851722	
glycine conjugate of C10H14O2 (1)		**0.8**	**0.72**	0.89		0.011098348	0.095583799	0.055017378	0.222567807	0.819664099	0.568388012	
branched-chain, straight-chain, or cyclopropyl 10:1 fatty acid (1)		**0.85**	0.94	**1.37**		0.034457902	0.13862355	0.435705267	0.426548407	0.074939513	0.188745601	
branched-chain, straight-chain, or cyclopropyl 12:1 fatty acid		0.63	**0.75**	1.03		0.170189507	0.327349933	0.023191077	0.185955146	0.767561062	0.548134677	
